# Temporal distribution of *Plasmodium falciparum* recrudescence following artemisinin-based combination therapy: an individual participant data meta-analysis

**DOI:** 10.1186/s12936-021-03980-z

**Published:** 2022-03-24

**Authors:** The WorldWide Antimalarial Resistance Network Methodology Study Group, Prabin Dahal, Julie Anne Simpson, Salim Abdulla, Jane Achan, Ishag Adam, Aarti Agarwal, Richard Allan, Anupkumar R. Anvikar, Emmanuel Arinaitwe, Elizabeth A. Ashley, Ghulam Rahim Awab, Quique Bassat, Anders Björkman, Steffen Borrmann, Teun Bousema, Hasifa Bukirwa, Verena I. Carrara, Marco Corsi, Michel Cot, Umberto D’Alessandro, Timothy M. E. Davis, Philippe Deloron, Meghna Desai, Pedro Rafael Dimbu, Djibrine Djalle, Abdoulaye Djimde, Grant Dorsey, Chris J. Drakeley, Stephan Duparc, Michael D. Edstein, Emmanuelle Espie, Abul Faiz, Catherine Falade, Caterina Fanello, Jean-Francois Faucher, Babacar Faye, Filomeno de Jesus Fortes, Nahla B. Gadalla, Oumar Gaye, J. Pedro Gil, Julius Gilayeneh, Brian Greenwood, Anastasia Grivoyannis, Tran Tinh Hien, Jimee Hwang, Bart Janssens, Elizabeth Juma, Erasmus Kamugisha, Corine Karema, Harin A. Karunajeewa, Jean R. Kiechel, Fred Kironde, Poul-Erik Kofoed, Peter G. Kremsner, Sue J. Lee, Kevin Marsh, Andreas Mårtensson, Mayfong Mayxay, Hervé Menan, Petra Mens, Theonest K. Mutabingwa, Jean-Louis Ndiaye, Billy E. Ngasala, Harald Noedl, Francois Nosten, Andre Toure Offianan, Bernhards R. Ogutu, Piero L. Olliaro, Jean Bosco Ouedraogo, Patrice Piola, Christopher V. Plowe, Mateusz M. Plucinski, Oliver James Pratt, Zulfikarali Premji, Michael Ramharter, Christophe Rogier, Lars Rombo, Philip J. Rosenthal, Carol Sibley, Sodiomon Sirima, Frank Smithuis, Sarah G. Staedke, Inge Sutanto, Ambrose Otau Talisuna, Joel Tarning, Walter R. J. Taylor, Emmanuel Temu, Kamala Thriemer, Nguyen Thuy-Nhien, Venkatachalam Udhayakumar, Johan Ursing, Michel van Herp, Marit van Lenthe, Michele van Vugt, Yavo William, Cornelis Winnips, Sophie Zaloumis, Issaka Zongo, Nick J. White, Philippe J. Guerin, Kasia Stepniewska, Ric N. Price

**Affiliations:** World Wide Antimalarial Resistance Network (WWARN), Oxford, UK

**Keywords:** Malaria, *Plasmodium falciparum*, Efficacy, Follow-up, Recrudescence, Distribution

## Abstract

**Background:**

The duration of trial follow-up affects the ability to detect recrudescent infections following anti-malarial treatment. The aim of this study was to explore the proportions of recrudescent parasitaemia as ascribed by genotyping captured at various follow-up time-points in treatment efficacy trials for uncomplicated *Plasmodium falciparum* malaria.

**Methods:**

Individual patient data from 83 anti-malarial efficacy studies collated in the WorldWide Antimalarial Resistance Network (WWARN) repository with at least 28 days follow-up were available. The temporal and cumulative distributions of recrudescence were characterized using a Cox regression model with shared frailty on study-sites. Fractional polynomials were used to capture non-linear instantaneous hazard. The area under the density curve (AUC) of the constructed distribution was used to estimate the optimal follow-up period for capturing a *P. falciparum* malaria recrudescence. Simulation studies were conducted based on the constructed distributions to quantify the absolute overestimation in efficacy due to sub-optimal follow-up.

**Results:**

Overall, 3703 recurrent infections were detected in 60 studies conducted in Africa (15,512 children aged < 5 years) and 23 studies conducted in Asia and South America (5272 patients of all ages). Using molecular genotyping, 519 (14.0%) recurrences were ascribed as recrudescent infections. A 28 day artemether-lumefantrine (AL) efficacy trial would not have detected 58% [95% confidence interval (CI) 47–74%] of recrudescences in African children and 32% [95% CI 15–45%] in patients of all ages in Asia/South America. The corresponding estimate following a 42 day dihydroartemisinin-piperaquine (DP) efficacy trial in Africa was 47% [95% CI 19–90%] in children under 5 years old treated with > 48 mg/kg total piperaquine (PIP) dose and 9% [95% CI 0–22%] in those treated with ≤ 48 mg/kg PIP dose. In absolute terms, the simulation study found that trials limited to 28 days follow-up following AL underestimated the risk of recrudescence by a median of 2.8 percentage points compared to day 63 estimates and those limited to 42 days following DP underestimated the risk of recrudescence by a median of 2.0 percentage points compared to day 42 estimates. The analysis was limited by few clinical trials following patients for longer than 42 days (9 out of 83 trials) and the imprecision of PCR genotyping which overcalls recrudescence in areas of higher transmission biasing the later distribution.

**Conclusions:**

Restricting follow-up of clinical efficacy trials to day 28 for AL and day 42 for DP will miss a proportion of late recrudescent treatment failures but will have a modest impact in derived efficacy. The results highlight that as genotyping methods improve consideration should be given for trials with longer duration of follow-up to detect early indications of emerging drug resistance.

**Supplementary Information:**

The online version contains supplementary material available at 10.1186/s12936-021-03980-z.

## Background

Before the introduction of molecular genotyping, the World Health Organization (WHO) recommended that anti-malarial clinical trials conducted in areas of high transmission should be restricted to a short follow-up duration (2 weeks). This was in part to reduce potential confounding from new infections which then could not be distinguished from recrudescences [1]. One of the earliest indications of emerging anti-malarial drug resistance is recrudescent parasitaemia [2]. Recrudescence is delayed until the anti-malarial drug no longer suppresses parasite multiplication. As resistance increases recrudescent parasitaemias occur as parasites are able to grow in increasing concentrations of the drug [2]. As the first recrudescences occur several weeks after treatment anti-malarial therapeutic efficacy trials with two weeks of follow-up could only identify high grade parasite resistance [2, 3].

In the 1990s, the introduction of polymerase chain reaction (PCR) genotyping allowed comparison of the sizes of amplified segments of highly polymorphic *Plasmodium falciparum* genes, paving the way for classifying probabilistically individual recurrent infections as either homologous (identical to the initial infection, i.e. recrudescent) or heterologous (different than the initial infection, i.e. a new infection) [4]. In 2003, the WHO revised its guidelines for assessing anti-malarial efficacy. The new guideline recommended 42 days of post-treatment follow-up for artemisinin-based combination therapy (ACT), including lumefantrine, and 63 days for artemisinin-based combinations including mefloquine, in conjunction with molecular genotyping to distinguish recrudescent from new infections [5, 6]. However conducting trials with such long follow-up was associated with significant logistical difficulties, and the guideline was revised again in 2009, to recommend a minimum of 28 and 42 days follow-up for artemisinin-based combinations including lumefantrine or mefloquine, respectively [7]. It was anticipated that the revised follow-up duration would capture “most” of the treatment failures, and thereby provide a reasonable approximation of drug efficacy.

Recent studies have reported that a substantial proportion of recrudescent infections emerge in the peripheral blood beyond the currently recommended follow-up duration. A Tanzanian study reported that 28 days of follow-up missed 28% (5/18) of recrudescent infections following treatment of 206 patients with artesunate-amodiaquine (ASAQ) and 58% (7/12) following treatment of 197 patients with artemether-lumefantrine (evaluable population) [8]. In an Ethiopian study, 88% (14/16) of the recrudescent infections were detected after 28 days following treatment with AL (n = 348 patients) [9]. Other studies have reported recrudescent infections occurring after 6 weeks after treatment with artesunate-mefloquine (ASMQ) and dihydroartemisinin-piperaquine (DP) [10–12], and up to 6 weeks after treatment with AL [12, 13]. Together, these reports suggest that the current recommendations regarding the duration of study follow-up warrant re-examination.

The aim of this study was to use pooled data from clinical trials to assess the ability of currently recommended minimum follow-up periods to capture PCR-confirmed recrudescence following treatment of uncomplicated *P*. *falciparum* malaria with fixed dose ACT.

## Methods

### Study and patient data

Clinical studies with fixed dose formulations of ACT uploaded in the WorldWide Antimalarial Resistance Network (WWARN) repository were selected if they had a minimum follow-up period of 28 days with molecular genotyping carried out to differentiate recurrent parasitaemia as due to either new infection or recrudescence [14]. Early treatment failures (on or before day 7) were excluded since the focus of the analysis was to characterize the temporal distribution of late recrudescences following initial parasite clearance. Patients with missing or indeterminate genotyping outcomes were also excluded. In Africa, analysis was restricted to children less than 5 years of age, since for this population immunity is predicted to have less impact on outcomes compared to older individuals. In the studies from Asia and South America, patients of all ages were included. Further details of study inclusion and exclusion criteria are presented in Additional file 1.

### The time of observed recrudescence

The time of observed recurrence ($$t_{obs} )$$ was used to estimate the time to recrudescence ($$T_{R} )$$ at which parasite recrudescence would exceed the threshold of detection (TOD); the latter was defined as 50 parasites per microlitre. $$T_{R}$$ was estimated assuming tenfold parasite multiplication per 48 h asexual cycle using the following algorithm [15]: $$\widehat{{T_{R} }} = t_{obs} - \left( {\left( {\log_{10 } (\frac{{P_{R} }}{50}} \right)} \right) \times 2)$$, where $$t_{obs}$$ is the day when recrudescence was detected in the study and $$P_{R}$$ is the parasite density at the observed time of recrudescence. If the estimated $$T_{R}$$ was less than the time of last visit when the patient had a negative peripheral blood film examination ($$t_{neg}$$), then $$T_{R}$$ was replaced as $$t_{neg} + 1$$.

### Estimation of temporal trend and distribution of recrudescence

The temporal trend (instantaneous risk) of recrudescence at any time during the study follow-up was quantified by its hazard function, $h\left( t \right)$. The adjusted estimate of the hazard function was derived from a Cox proportional hazard model controlling for the body weight adjusted (mg/kg) dose of the partner drug, age of the patient, and initial parasite load (on log-scale). The baseline hazard function $h_{0} \left( t \right) {\text{was}}$ approximated as the slope of the cumulative baseline hazard $(H_{0} \left( t \right))$. $H_{0} \left( t \right)$ was estimated using fractional polynomial smoothing after applying log-transformation as outlined previously [16]. The fractional polynomial approach was used, as standard parametric approaches were found to provide a poor fit to the data (Additional file 1; Sections 2 and 3). The probability density $f_{0} \left( t \right)$ of time to PCR confirmed recrudescence was then estimated as $f_{0} \left( t \right) = h_{0} \left( t \right).S_{0} \left( t \right)$, where $S_{0} \left( t \right)$ is the baseline survival estimate of drug efficacy ($S_{0} \left( t \right) = e^{{ - H_{0} \left( t \right)}}$). The constructed distribution was normalized such that the area under the curve was equal to 1. The area under the normalized probability density curve (AUC) was calculated at a given time-point and the cumulative AUC at a time-point $t$ was reported with the associated 95% confidence interval (CI) derived using 1000 bootstrap resamples drawn from data of the same sample size. The proportion of recrudescences missed at any specific time point was calculated as 1—AUC at that time-point. For the DP regimen, the distribution was estimated for those who received a total piperaquine dose of above or below 48 mg/kg, a threshold associated with poorer therapeutic outcomes in a paediatric population [17]. For AL, the derived estimates were stratified by region as suggested previously [18].

### Simulation studies

The impact of a missed proportion of recrudescences on the absolute bias (overestimation) in a derived estimate of efficacy was evaluated through two simulation studies. The first simulation study explored the impact of sub-optimal follow-up on derived efficacy in areas of low and high malaria transmission, and Kaplan–Meier estimates were generated at the end of the maximum follow-up time (day 63) and compared with the estimates derived for days 28 and 42 (Additional file 1: Section 4). The second simulation study explored how the duration of follow-up influenced the estimated efficacies of AL and DP. Hazard ratios were estimated using Cox proportional hazards regression at days 28, 42 and 63. Summarized results were reported from 1000 simulation runs. The design of the simulation studies has been described previously [19] and a step-by-step outline of the simulation protocol is presented in supplemental text (Additional file 1: Sections 4).

### Sensitivity analyses

The following two sensitivity analyses were considered: (i) distributions were re-estimated by using data only from studies with 42 days of follow-up, and (ii) the 707 patients with indeterminate recurrence excluded in the primary analysis were considered as missing data, and the probability distributions were re-estimated using multiply imputed data, as previously described [20].

### Software

Cumulative baseline hazard was estimated by fitting the Cox proportional hazard model using the *survival* library; Kaplan–Meier type hazard and kernel-smoothed hazard function were estimated using the *muhaz* library in R software [21].

### Results

**Fig. 1:**
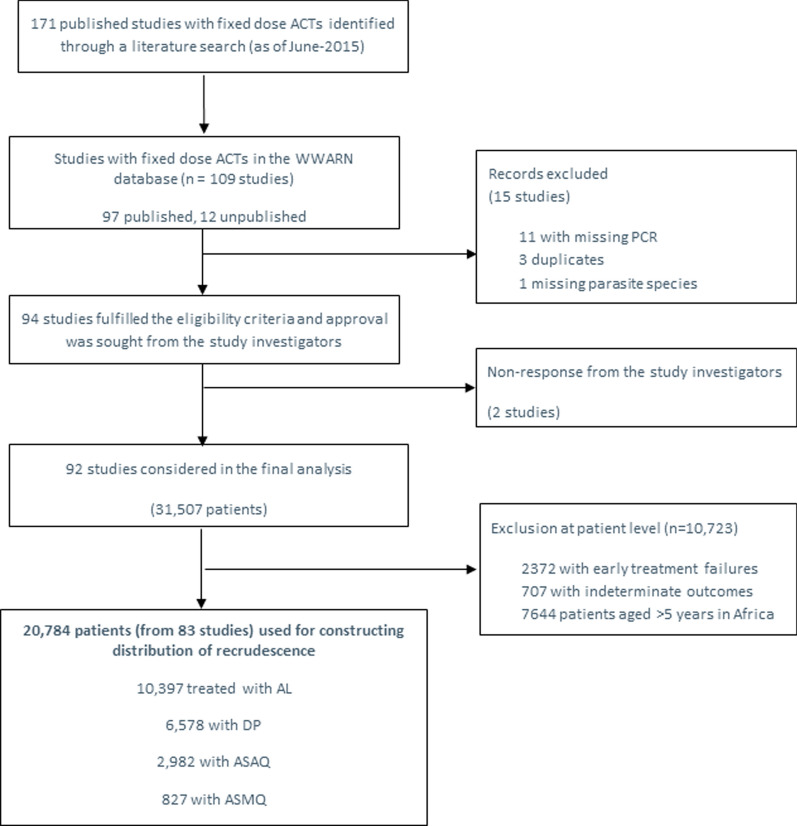
Flow chart showing selection of clinical trials and participants in the study. AL = artemether-lumefantrine; DP = dihydroartemisinin-piperaquine; ASMQ = artesunate-mefloquine; ASAQ = artesunate-amodiaquine, and n = number of recrudescences; PCR = Polymerase Chain Reaction; WWARN = WorldWide Antimalarial Resistance Network; ACT = artemisinin-based combination therapy

Data were available for 15,512 children aged less than 5 years from 60 studies in Africa and for 5272 patients of all ages from 23 studies in Asia and South America (Fig. 1). The duration of follow-up was 28 days in 39 (47.0%) studies, 42 days in 32 (38.6%) studies, 56 days in 3 (3.6%) studies and 63 days in 9 (10.8%) studies. The studies were published from 2001 to 2015 with 45 (54.2%) studies published before 2010. Further details on the study designs and patient characteristics are provided in Additional file 1.

### Recurrent parasitaemia and molecular genotyping

A total of 3703 (17.8%) patients had recurrent parasitaemia between days 7 and 63 after initiation of treatment. Parasite genotyping was attempted at three or more loci (including microsatellites) in 3026 (81.7%) recurrent infections, 2 loci in 575 (15.5%) infections, one locus in 79 (2.1%) infections, and using microsatellites only in 15 (0.4%) infections. The number of markers was not documented in 8 (0.2%) infections. In total 3184 (86.0%) recurrent infections were classified as new infections and 519 (14.0%) as recrudescent infections. Among recurrent infections, the proportion of recrudescent infections was 20.3% (16/79) in studies which used a single marker, 16.5% (95/575) in studies with two markers, 13.2% (399/3026) in studies with three markers (including microsatellites), 33.3% (5/15) in studies using only microsatellites, and 50% (4/8) in studies with no description of molecular methods.

### Temporal trend of *P. falciparum* recrudescence with AL and DP

**Fig. 2:**
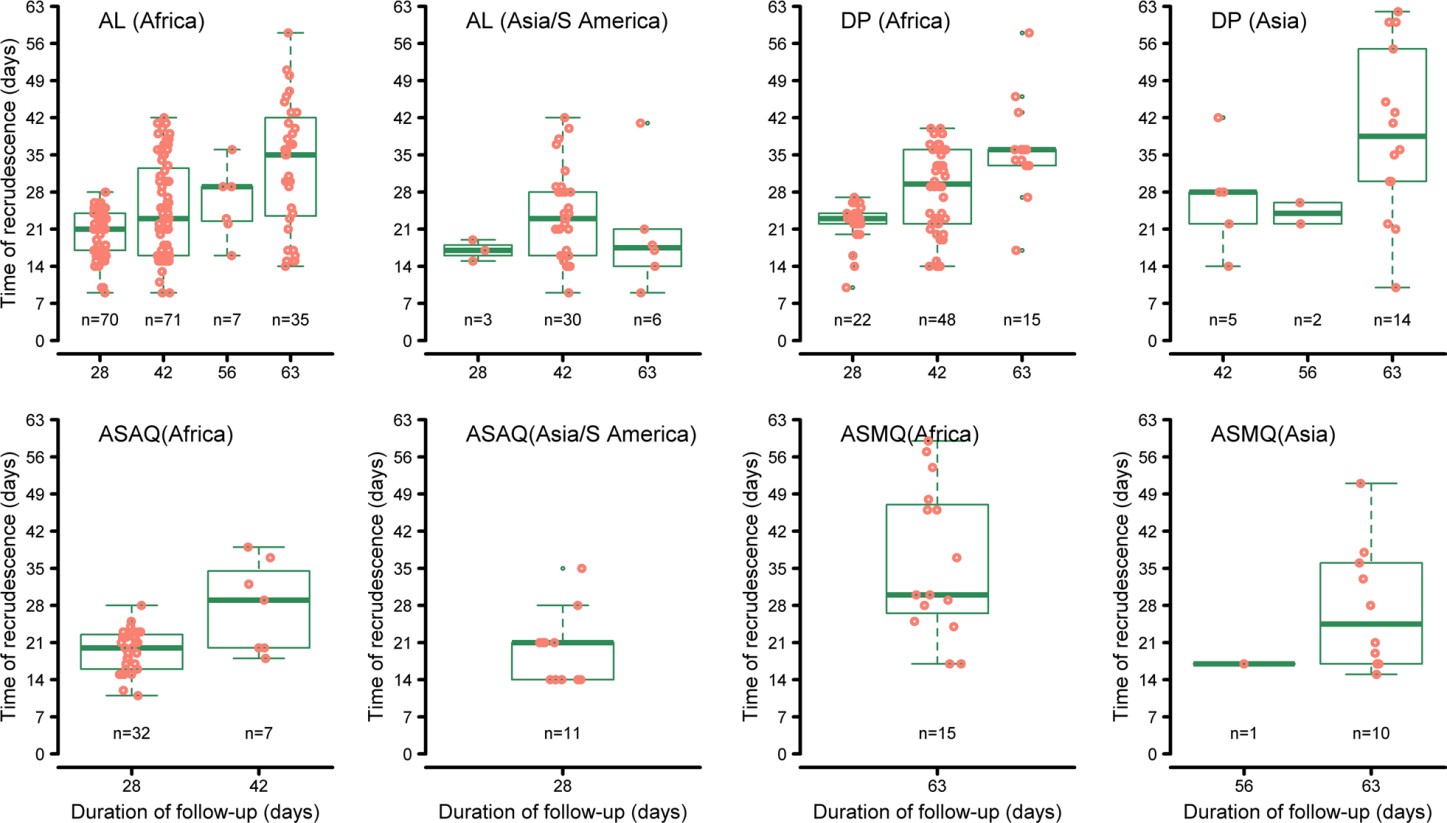
Observed time-to-recrudescence for ACT stratified by region and duration of follow-up. The y-axis depicts the time for the recrudescent infection to reach microscopic limit of detection (50 parasites/µL). In Africa, the distribution is shown for children < 5 years age, whereas in Asia/ S. America, data are shown for patients of all ages. Each dot represents an observed recrudescence. AL = artemether-lumefantrine; DP = dihydroartemisinin-piperaquine; ASMQ = artesunate-mefloquine; ASAQ = artesunate-amodiaquine, and n = number of recrudescences. Only data from studies with at least three molecular markers are shown in the graph. For DP (Asia), the graph depicts recrudescences observed in studies from Asia and S. America combined

**Fig. 3:**
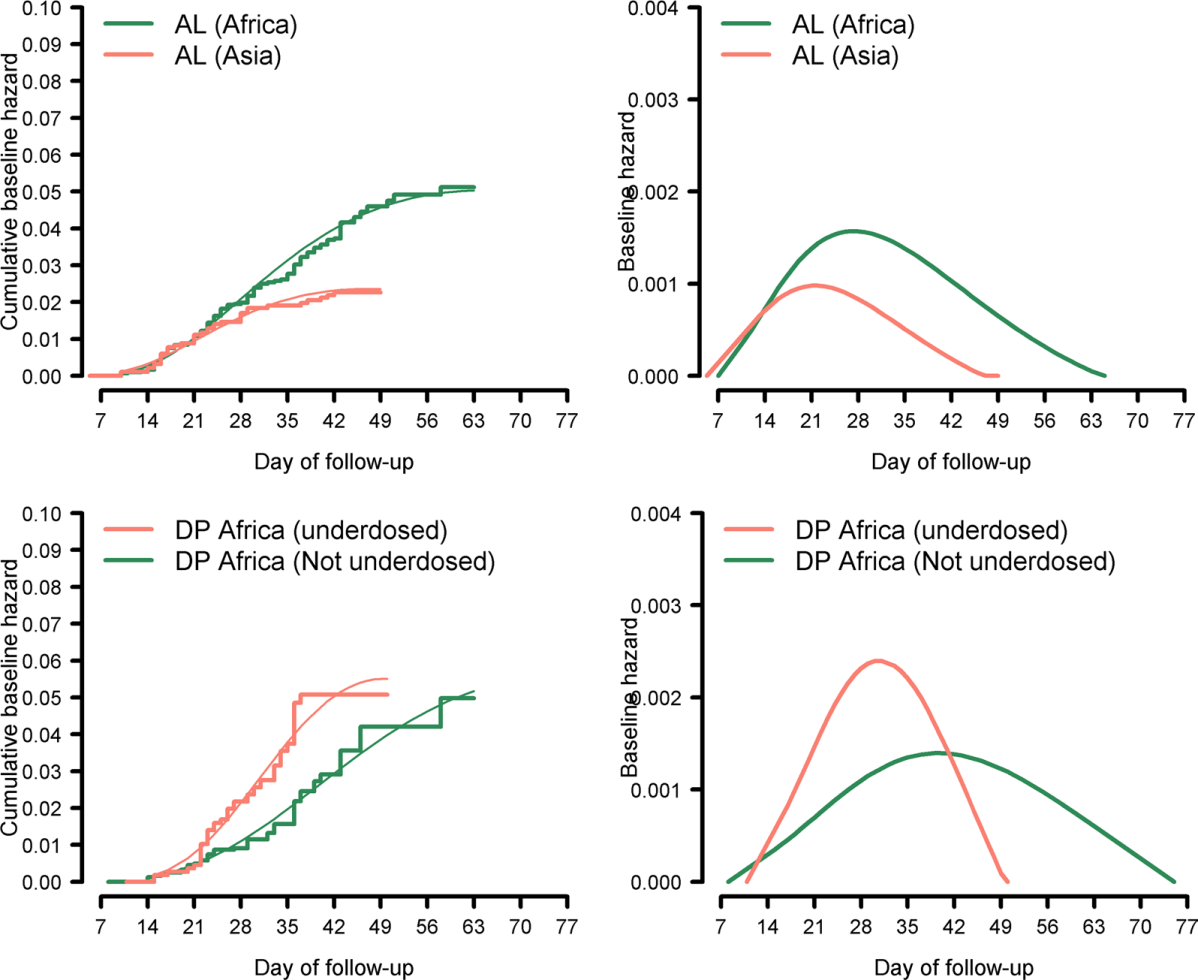
Fractional polynomial estimates of cumulative and instantaneous hazard for AL and DP. The cumulative baseline hazard estimated from Cox model (adjusted for age, baseline parasitaemia and mg/kg dosage of partner drug) together with fractional polynomial smoother (left panel). The temporal trend of observing recrudescence during the follow-up period, estimated by the instantaneous baseline hazard function (right panel). Data in Africa was restricted to children < 5 years whereas patients of all ages were included in Asia and S. America. Data from S. America were grouped with Asia. All studies used at least 3 locus genotypes (the usage of microsatellites was considered as a separate locus). Under-dosed was defined as total piperaquine dose ≤ 48 mg/kg (those receiving > 48 mg/kg defined as not under-dosed). AL = artemether-lumefantrine; DP = dihydroartemisinin-piperaquine. The equations for the cumulative baseline hazard functions derived using the fractional polynomial smoothing are presented in the Additional file 1

A total of 306 recrudescent infections were confirmed after AL treatment (264 in Africa; 41 in Asia and 1 in South America) and 115 after DP treatment (94 in Africa; 19 in Asia, and 2 in South America). The observed times of recrudescence in different regions stratified by follow-up duration are presented in Fig. 2. The non-parametric estimates and the kernel smoothed estimate of the hazard function exhibited a non-monotonic trend (Additional file 1: Section 2). Covariate adjusted smoothed hazard functions obtained from Cox models exhibited a non-monotonic shape for AL and DP (Fig. 3; right panels). For AL, the instantaneous hazard function was shifted to the right for Africa compared to Asia, and the peak hazard was lower and earlier in Asia (day 21) compared to Africa (day 27). For DP, the peak hazard was earlier (day 30) in those who were under-dosed (< 48 mg/kg of piperaquine) compared to those who received a higher dose of piperaquine (day 39). There were a total of 72 recrudescent infections following treatment with fixed dose formulations of ASAQ (61 in Africa and 11 in Asia/S.America) and 26 (15 in Africa and 11 in Asia) following treatment with fixed dose ASMQ; these low numbers precluded robust modelling for these regimens. These patients were excluded in the construction of distribution of failure times.

### The empirical distribution of time of *P. falciparum* recrudescence

**Fig. 4:**
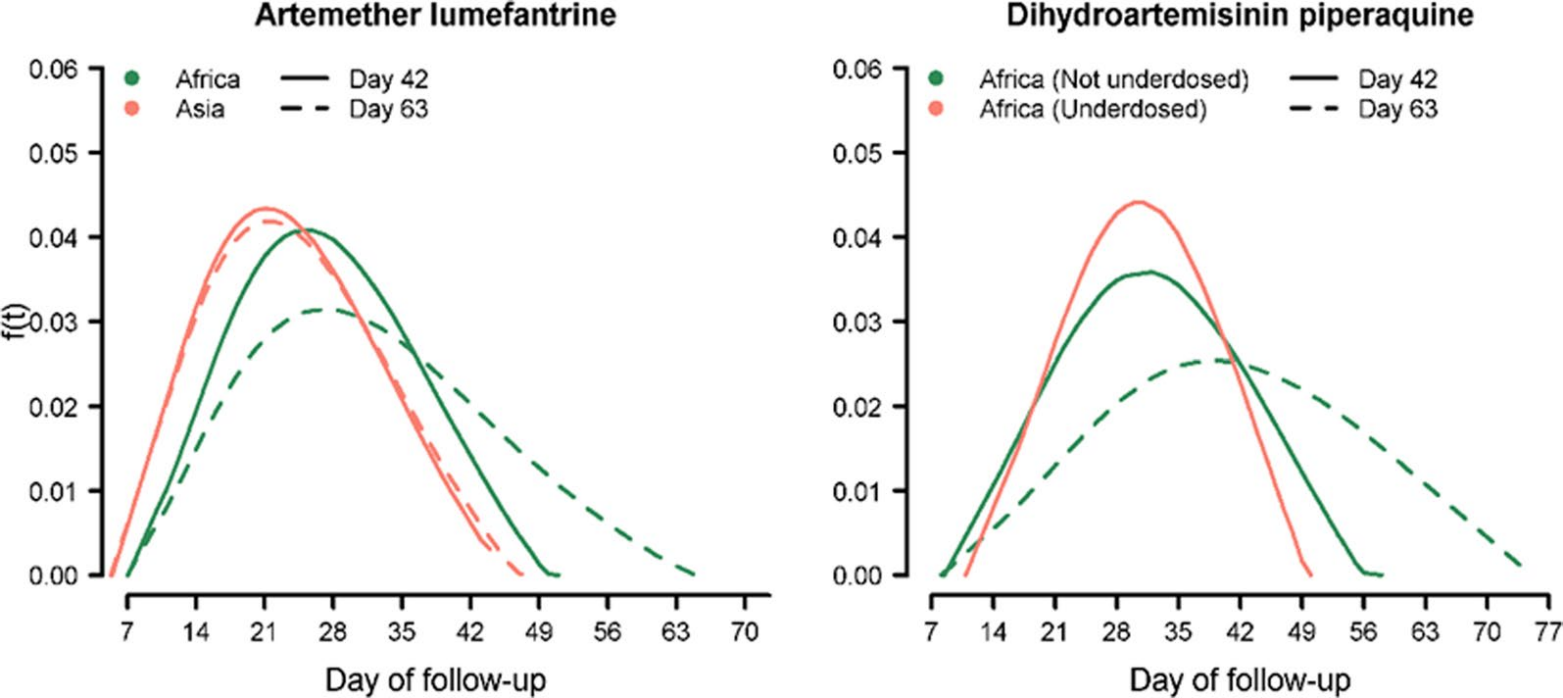
Distribution of recrudescent infection for artemether-lumefantrine and dihydroartemisinin-piperaquine. Dotted line represents distribution derived from all data. Solid line represents the distribution derived by including data up to day 42 (observations beyond day 42 were censored on day 42). For Africa, the data included children < 5 years old whereas for Asia, no age restriction was applied

The probability density of the time to recrudescence in patients treated with AL and DP regimens is presented in Fig. 4. Overall the area under the curve (AUC) for AL in Africa was 0.42 [95% CI 0.26–0.53] on day 28, 0.79 [95% CI 0.53–0.97] on day 42 and 1.00 [95% CI 0.81–1.00] on day 63 (Table 1). In Asia, the distribution was shifted to the left compared to that observed in Africa, with an estimated AUC of 0.68 [95% CI 0.55–0.85] on day 28 and 0.98 [95% CI 0.93–1.00] on day 42. The distribution of recrudescence following DP could only be derived from African studies in which 85 of the 514 recurrences were categorized as recrudescence by PCR. In Asia/S. America, only 21 of 206 recurrences were categorized as recrudescences. The distribution of recrudescence following DP in Africa was shifted to the left in those who were under-dosed (receiving ≤ 48 mg/kg); the AUC by day 42 was 0.91 [95% CI 0.78–1.00] in the under-dosed group compared to 0.53 [95% CI 0.10–0.81] in those treated with a higher dose (Table 1).

**Table 1:** The area under the curve of the estimated probability distribution

Day	AL in Africa^a^ 95% CI]	AL in Asia^a^ [95% CI]	DP in Africa (PIP mg/kg ≤ 48) [95% CI]	DP in Africa (PIP mg/kg > 48) [95% CI]
28	0.42 [0.26–0.53]	0.68 [0.55–0.85]	0.39 [0.25–0.52]	0.20 [0.00–0.36]
35	0.63 [0.40–0.78]	0.88 [0.78–0.99]	0.69 [0.53–0.83]	0.36 [0.03–0.59]
42	0.79 [0.53–0.97]	0.98 [0.93–1.00]	0.91 [0.78–1.00]	0.53 [0.10–0.81]
49	0.91 [0.64–1.00]	1.00 [0.93–1.00]	1.00 [0.93–1.00]	0.70 [0.22–0.99]
56	0.97 [0.74–1.00]	1.00 [0.94–1.00]	1.00 [0.98–1.00]	0.84 [0.36–1.00]
63	1.00 [0.81–1.00]	1.00	1.00	0.93 [0.47–1.00]

AL, artemether-lumefantrine; DP, dihydroartemisinin-piperaquine; PIP, piperaquine; CI, confidence interval

^a^Estimated in children < 5 years in Africa and in patients of all ages from Asia

The 95% confidence interval was estimated from 1000 bootstrap samples of data for AL; for DP not under-dosed this was from 925 samples, and for DP under-dosed this was based on 987 bootstrap samples drawn from the original dataset

### Simulation studies

**Fig. 5:**
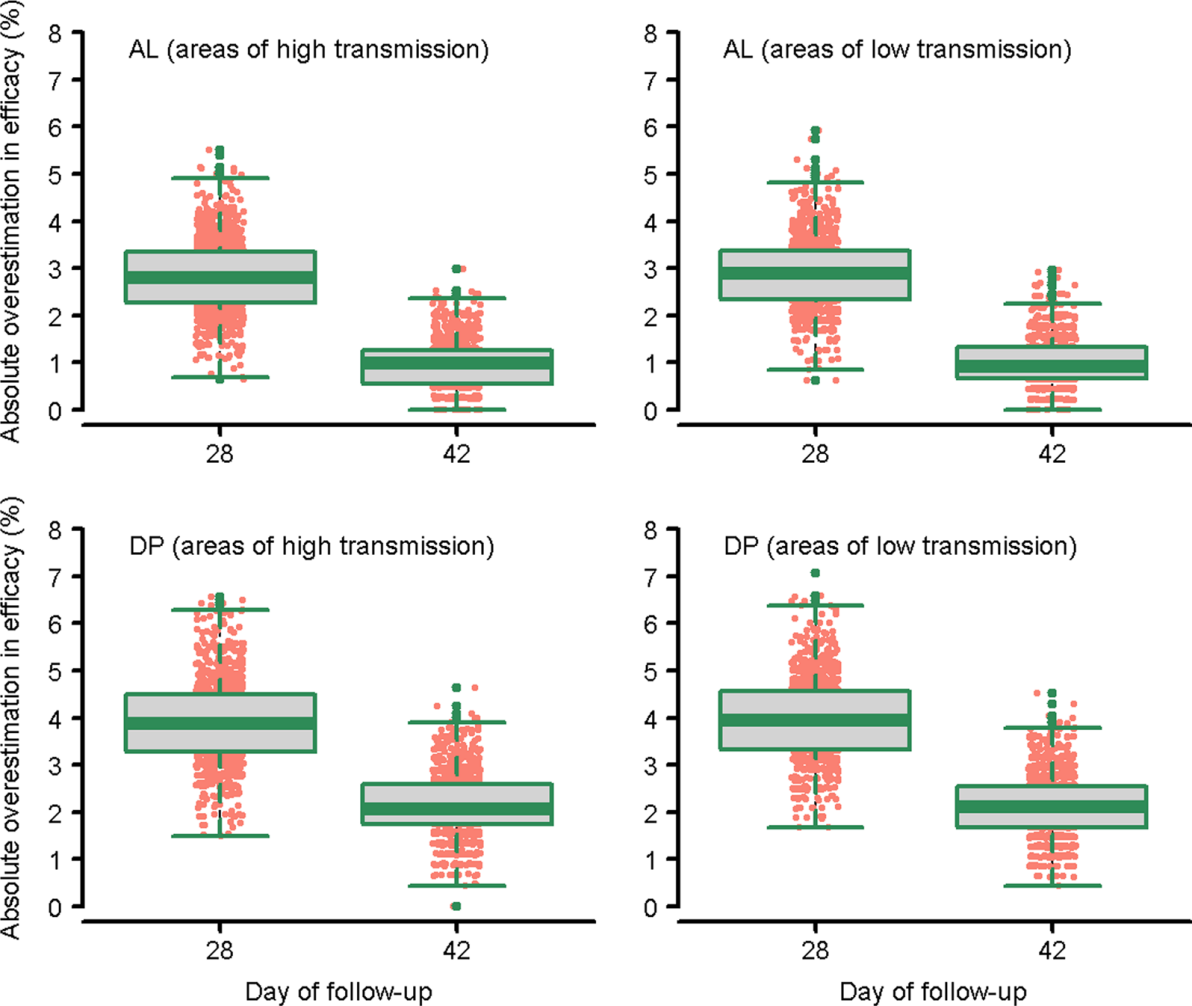
Absolute overestimation in efficacy due to sub-optimal follow-up duration (compared to day 63 estimates). Overestimation of efficacy relative to day 63 estimates for AL and DP regimen. In areas of high transmission, there was a median of 15% new infections in the DP arm and 30% new infections in AL arm representing. In areas of low transmission, there was a median of 8% new infections in the DP arm and 15% new infections in the AL arm. Simulation assumed 500 patients per treatment arm and was repeated for 1,000 runs. AL = artemether-lumefantrine; DP = dihydroartemisinin-piperaquine (See Additional file 1 for further details on the functions used for simulation)

Simulation studies were undertaken to assess correlations between duration of follow-up and estimates of drug efficacy. Compared to the “true” efficacy estimates of AL as measured at day 63, estimates derived using only the recrudescence observed until day 28 overestimated the drug efficacy by a median of 2.8 percentage points [Interquartile Range (IQR): 2.3%–3.4%; Range: 0.6%–5.5%] in areas of high transmission. With 42 days of follow-up, the overestimation in efficacy fell to 0.9 percentage points [IQR: 0.7%–1.3%; Range: 0.0%–2.9%] (Fig. 5; top-left panel). Compared to the true efficacy estimates for DP as measured at day 63, those generated at day 42 overestimated drug efficacy by 2.0 percentage points [IQR: 1.7%–2.6%; Range: 0.0%–4.6%] in high transmission settings (Fig 5; bottom left panel). The estimates for areas of low transmission intensity were similar (Fig. 5; right panels).

### Impact of follow-up duration on comparative efficacy

**Fig. 6:**
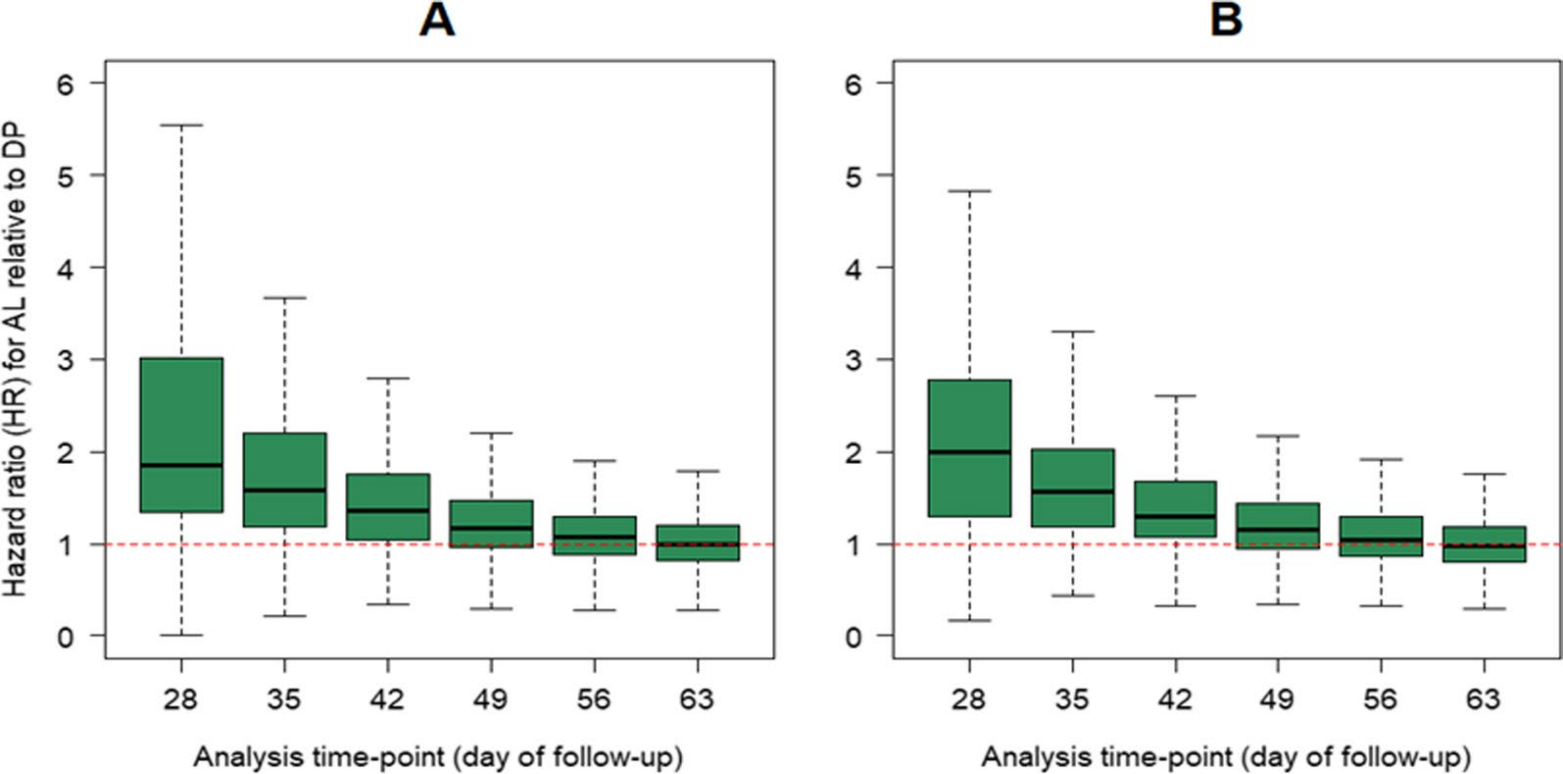
Simulation study comparing hazards ratio of recrudescence for AL against DP in children < 5 years in Africa. **A** There were a median (across 1000 simulation runs) of 8% new infections in DP arm and 15% in AL arm representing areas of low transmission. **B** There were a median of 15% new infections in DP arm and 30% in AL arm representing areas of high transmission. In both simulation settings, approximately 4% recrudescence was observed by day 63 on AL and DP arm. Data for AL was simulated based on the estimated hazard function of recrudescence in children < 5 years Africa, and for DP regimen the data was simulated based on the hazard function for those who received piperaquine dose greater than 48 mg/kg (See Additional file 1 for the functions used for these simulations). The dotted horizontal line shows the line of no effect (hazard ratio = 1). The simulation assumed 500 patients per treatment arms. AL = artemether-lumefantrine; DP = dihydroartemisinin-piperaquine

The effect of study duration on comparative efficacy in randomized comparative studies was investigated. In areas of low transmission in which the risk of new infections was low (8% in the DP arm and 15% in the AL arm), the median hazard ratio (HR) for AL relative to DP (for recrudescence) was 2.00 [IQR: 1.29–2.78] at day 28, 1.30 [IQR: 1.07–1.68] on day 42 and fell to parity from day 49 onwards (Fig. 6; left panel). The results were similar in areas of high transmission (Fig. 6; right panel). There was little difference in the results when the simulation was repeated with 200 and 1000 subjects per treatment arm (Additional file 1: Section 4).

### Sensitivity analyses

When the analysis was restricted to outcomes at day 42 and observations beyond this time-point were censored, the distribution of the timing of recrudescence after AL in Africa shifted to the left compared to the distribution including all data up until day 63 (Fig. 4; left panel, solid lines). Similarly, the distribution of recrudescence for DP in those treated with ≥ 48 mg/kg also shifted to the left when the analysis was restricted to day 42 (Fig. 4; right panel, solid lines). These sensitivity analyses remained unchanged when indeterminate outcomes were considered as missing data (Additional file 1: Section 5).

## Discussion

Using data from 83 anti-malarial clinical trials conducted in Asia, Africa and South America, this analysis characterized the temporal and cumulative distributions of recrudescence after treatment with AL or DP in patients with uncomplicated *P. falciparum* malaria. The temporal trend of *P. falciparum* recrudescent parasitaemia exhibited a non-monotonic shape, an observation consistent with anti-malarial pharmacokinetics. Soon after therapy the risk of recrudescence is extremely low, as most parasites are rapidly cleared by the highly potent artemisinin components of the ACT. Subsequently, as concentrations of the partner drug fall below the minimum inhibitory concentration (MIC), any remaining parasites can grow and expand, eventually leading to patent infection and symptomatic illness; during this period, the instantaneous risk of recrudescence rises. In a population of patients the hazard of recrudescence rises as blood concentrations of the antimalarial drug fall below MIC values and then falls again as the majority of recrudescences have occurred. New infections are also constrained by residual concentrations of the slowly eliminated antimalarial drugs and “bunch” together after blood levels fall below prevailing MIC values [22].

Characterization of the temporal trend enabled the construction of the probability distribution of the infections estimated to be recrudescences. In African children less than 5 years old treated with DP, the peak distribution of recrudescence was around day 39 after initiation of therapy in children who were adequately dosed (≥ 48 mg/kg total dose of piperaquine), but this fell to day 30 in children treated with a lower total dose of piperaquine (< 48 mg/kg). This is consistent with patients treated with a lower dose having blood concentrations which fall below the MIC sooner, allowing the parasite biomass to become patent earlier.

In patients treated with AL there were significant regional differences in the probability distributions of recrudescent infections with a shift to the left in Asia and South America (in patients of all ages) compared to Africa (in children < 5 years). This may reflect suppression of the parasite growth by host immunity, which is acquired earlier in life in Africa, where children often have multiple infections per year, whilst immunity is acquired more slowly in low endemic settings in Asia and South America [23]. However this explanation does not explain adequately why that suppression should then decrease to allow recrudescence. The derived distribution of recrudescences in high transmission settings, such as Africa, needs to be interpreted with caution, as late recrudescence beyond day 42 may also have arisen through misclassification of new infections as recrudescences. In higher transmission settings, the proportion of recurrent infections that are due to reinfection increases in studies with longer follow up, until eventually all recurrent infections are newly acquired. The uncertainty is particularly pertinent for individuals treated with AL; only a small proportion of patients (11.4%) were followed for more than 42 days and the infections ascribed as recrudescences detected between days 42 and 63 in 9 patients influenced the estimation (See Fig. 4 on the impact of follow-up duration on the estimated distribution) [12, 24]. In areas of high transmission polyclonal infections are common and these confound the interpretation of genotyping data [25, 26]. In multilocus genotyping using separate PCRs the amplified sequences are not phased so haplotypes cannot be inferred if there are multiple genotypes. The proportion of recurrent infections can be overestimated or underestimated depending on the background prevalence and distribution of the polymorphic markers, the technique used, the criteria set, and the number of reinfections (transmission intensity). In the one African study of AL, in which patients were followed until day 84, recrudescence was defined as persistence of at least one baseline clone, and this is likely to have led to a substantial proportion of late new infections being misclassified as recrudescent infections [24]. When the distributions of recrudescence following AL in Africa were derived using only data from studies with 42 days follow up the distribution shifted to the left and this was far more apparent in Africa than Asia (Fig. 4).

To estimate the degree to which absolute efficacy might be overestimated, simulation studies for AL and DP regimens were undertaken. Compared to the efficacy estimates at day 63, studies of AL restricted to 28 days follow-up overestimated drug efficacy by a median of 2.8% (range: 0.6%–5.5%), whereas studies of DP restricted to 42 day follow-up overestimated efficacy in adequately dosed DP by a median of 2.0% (range: 0%–4.6%). These results suggest that follow-up of 28 days for AL and 42 days for DP may result in failure to detect early signs of recrudescent parasitaemia occurring after the end of follow-up. Overall, the impact of follow-up on estimated treatment efficacy was modest, and any additional gain in accuracy in deriving these estimates must be weighed against the increased logistical challenge of studies with longer follow-up and a higher risk of misclassification when defining recrudescences and reinfections.

Recrudescent parasitaemia is the primary determinant for the selection and onward transmission of de novo resistant parasites [15]. Such selection and propagation of resistance is predicted to occur within a “window” determined by a drug’s pharmacokinetic profile [27]. For the standard AL regimen, this hypothetical window lies between days 24 to 27 for emergence of de novo resistance and between days 20 to 39 for acquired resistance [27]. The corresponding window for mefloquine (a drug with a longer elimination half-life) is estimated to be between 73 to 87 days and 65 to 113 days for the emergence of de novo and acquired resistance, respectively [27].

The analysis presented has a number of important limitations. First, the estimation of the hazard function is vulnerable to the method used for estimation, especially on the distal part of the distribution (See Additional file 1: Section 2). Second, only 9 studies included in the analysis had a follow-up greater than 42 days and there were only 255 recurrent events (25 recrudescences and 230 new infections) detected beyond this period, affecting the tail-end of the derived distributions. When the analysis was restricted to studies with only 42 days of follow-up, the estimated distribution of recrudescence shifted to the left compared to that derived using all available data (Fig. 4; solid lines). Despite the shifts in the distributions, the results indicated that the currently recommended follow-up times do not capture all late recrudescence. Third, the definition of recrudescence is dependent upon the molecular methods applied and their interpretation. Current genotyping methods are imprecise and, in areas of high transmission, overcall late recrudescent infections [25]. Whilst data on the number of polymorphic loci used to genotype were documented in all but 8 of the 3026 recurrent infections, no data were available on the population allele frequencies of these markers or the multiplicity of infection, and hence it was not possible to discern the degree to which late recrudescing parasites may have been misclassified. This will have affected the tail of the temporal distribution, particularly in high transmission settings, where reinfection is common. Accounting for this error, when constructing the empirical distribution and in the simulation studies, was beyond the scope of this work, and hence the tail of the distribution of recrudescence following AL in Africa should be interpreted with caution. Finally, the estimated time of recrudescence assumed tenfold parasite multiplication per 48-h asexual cycle for estimating the time when the parasite density would have first reached 50 parasites/µl, and yet this is likely affected by host characteristics, such as acquired immunity, and by circulating drug concentrations, which could not be quantified in this analysis [15, 28].

## Conclusions

The derived empirical distribution of recrudescence indicates that the currently recommended minimum follow-up for anti-malarial efficacy trials do not capture a significant proportion of PCR confirmed recrudescences occurring after AL and DP treatment. Whilst the overall impact of this limitation on the estimated efficacy of these anti-malarial regimens was modest in absolute terms, extension of the duration of follow-up to 42 days for AL and 63 days for DP, particularly with more precise methods of genotyping, would facilitate detection of early signs of emerging drug resistance, which can manifest through delayed parasite recrudescence.

## Supplementary Information

**Additional file 1.** Further methodological details and results.

**Additional file 2.** List of studies used.

## Data Availability

The data included during in the current study is available from the WorldWide Antimalarial Resistance Network (WWARN), a controlled open access repository of anti-malarial studies, on reasonable request through the Data Access Committee: http://www.wwarn.org/about-us/governance-people/data-access-committee.

## Abbreviations

ACT: Artemisinin-based combination therapy
AL: Artemether-lumefantrine
ASAQ: Artesunate-amodiaquine
ASMQ: Artesunate-mefloquine
AUC: Area under the density curve
CI: Confidence interval
DP: Dihydroartemisinin-piperaquine
HR: Hazards ratio
PCR: Polymerase chain reaction
PIP: Piperaquine
WWARN: WorldWide Antimalarial Resistance Network
