# Supplementary material for: Temporal distribution of Plasmodium falciparum recrudescence following artemisinin-based combination therapy: an individual participant data meta-analysis
Source: Malar J. 2022 Mar 24;21:106. doi: 10.1186/s12936-021-03980-z (PMC8943927; doi:10.1186/s12936-021-03980-z)
Supplement: Supplementary file 1 — Additional file 1. Further methodological details and results. [file 12936_2021_3980_MOESM1_ESM.docx]

**Additional file 1:**

**Temporal distribution of *Plasmodium falciparum* recrudescence following artemisinin combination therapies: An individual participant data meta-analysis**

The WorldWide Antimalarial Resistance Network Methodology Study Group ^1^

^1^WorldWide Antimalarial Resistance Network (WWARN), Oxford

**Correspondences**:

[prabin.dahal@wwarn.org](mailto:prabin.dahal@wwarn.org)

ric.price@wwarn.org

## Selection of studies for inclusion

## Data selection from the WorldWide Antimalarial Resistance repository (WWARN)

Study were selected for this analysis if they satisfied the following inclusion and exclusion criteria:

## Inclusion & Exclusion criteria

Data in the WWARN repository were eligible for inclusion if they fulfilled the following criteria:

1. Prospective clinical efficacy studies of uncomplicated *P. falciparum* (alone or mixed infections with *P. vivax*)
2. Clinical trials with one of the following fixed dose combination of artemisinin combination therapies
   1. artemether-lumefantrine (AL)
   2. dihydroartemisinin-piperaquine (DP)
   3. artesunate-amodiaquine (ASAQ)
   4. artesunate-mefloquine (ASMQ)
3. Polymerase Chain Reaction (PCR) genotyping performed to distinguish recrudescence from new infection using all or some of the molecular markers merozoite surface protein (*msp)-1, msp-2,* or glutamate rich protein *(glurp*)
4. A minimum follow-up duration of 28 days

Studies with only fixed dose combination of the ACTs were considered as it has been shown to provide optimal dosing compared to the loose tablets for the combination ASAQ [1]. Similarly, the loose ASMQ combination varies substantially with regards to the target dose of mefloquine administered; hence the screening was restricted to the fixed dose combination of this regimen. Studies on prophylaxis, severe malaria, pregnant women, and patients with known HIV co-infection, hyperparasitaemia, studies in healthy volunteers and travelers, and pure *P. vivax* malaria formed the exclusion set.

## Desirable criteria

- Outcome classification based on each of the individual genotyping markers including the length of pre-and post-recurrence samples for each of the allelic family
- Multiplicity of infection - the number of different *P. falciparum* strains co-infecting a single host

## Studies included

A database search restricted to the WWARN repository was carried out to identify studies fulfilling the inclusion criteria outlined in section 1.1 (dated 06-June-2015). A total of 109 studies with fixed dose formulation of artemisinin combination therapies (ACTs) were identified, of which 15 didn’t meet the inclusion criteria (figure 1). An email was sent to the principal investigators (and the corresponding authors) of the 94 eligible studies requesting permission to use these studies for this methodological work. A brief outline of the aims and objectives of the DPhil study was sent along with the email request. In two studies (both were from the same investigator) no response was received and a total of 92 studies where permission was granted formed the study database. After restricting the patient population to children <5 years in Africa and patient of all ages in Asia/S. America, there were 83 studies eligible for inclusion (Table 1).

Details of the studies included are provided in Table 1.

Figure 1 shows the location of sites where the studies were conducted for each of the drug regimen.


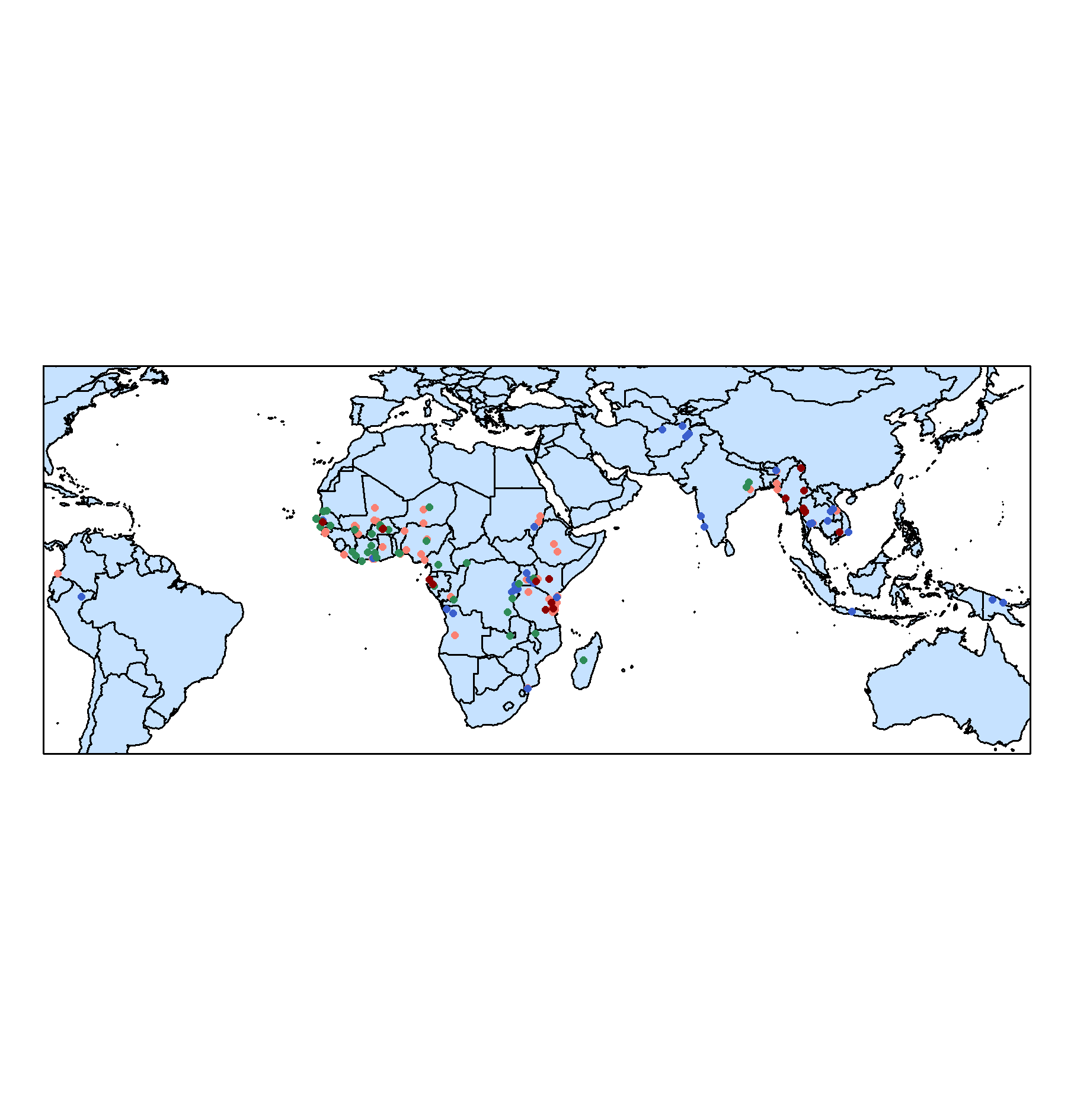


Figure S1: Study site locations for different drugs

Legend: •artesunate-mefloquine; •artemether-lumefantrine; •artesunate-amodiaquine; •dihydroartemsinin-piperaquine

**Table S1: Characteristics of the studies included**

| **WWARN ID** | **Study** | **N** | **Region** | **Year of enrolment** | **Age range**  **(months)** | **Follow-up**  **duration** | **Genotyping**  **markers** | **Treatment**  **Supervision** | **Randomisation** |
| --- | --- | --- | --- | --- | --- | --- | --- | --- | --- |
| ADXZX | Bouyou-Akotet-2010 [2] | 41 | Africa | 2005—2005 | 12—72 | 28 | *msp-1, msp-2* | Full | Yes |
| AJXCU | Menan-2011 [3] | 206 | Africa | 2008—2009 | 24—744 | 28 | *msp-1, msp-2* | Full | Yes |
| AKABP | Mutabingwa-2005 [4] | 285 | Africa | 2002—2003 | 4—59 | 28 | *msp-2* | Unsupervised | Yes |
| ATMFH | Tran-2012 [5] | 55 | Asia | 2010—2011 | 120—780 | 42 | *msp-1, msp-2, glurp* | Full | Yes |
| CCEPC | Piola-2005 [6] | 953 | Africa | 2002—2004 | 12—960 | 28 | *msp-1, msp-2, glurp* | Full/Partial | Yes |
| CDCMJ | Smithuis-2010 [7] | 400 | Asia | 2008—2009 | 12—792 | 63 | *msp-1, msp-2, glurp* | Unsupervised | Yes |
| CDTEN | de Wit-2015* | 283 | Africa | 2013—2014 | 0—252 | 42 | *msp-1, msp-2* | Full | Yes |
| CRAXS | Thanh-2009 [8] | 60 | Asia | 2006—2007 | 72—720 | 42 | *msp-1, msp-2* | Full | Yes |
| CUDNY | Janssens-2007 [9] | 182 | Asia | 2002—2003 | 24—780 | 63 | *msp-1, msp-2, glurp* | Full | Yes |
| CXJYT | Falade-2005 [10] | 310 | Africa | 2002—2003 | 2—119 | 28 | *msp-1, msp-2, glurp* | Full | Non |
| DAABT | Laminou-2011* | 159 | Africa | 2011—2011 | 2—168 | 28 | *msp-1, msp-2, glurp* | Partial | Yes |
| DADPZ | Arinaitwe-2009 [11] | 232 | Africa | 2007—2008 | 45—221 | 28 | *6 microsatellites* | Partial | Yes |
| DAXCM | Sawa-2013 [12] | 298 | Africa | 2009—2009 | 12—120 | 42 | *msp-1, msp-2* | Full | Yes |
| DBCXT | Clark-2010 [13] | 144 | Africa | 2004—2008 | 13—144 | 28 | *msp-1, msp-2,*  *4 microsatellites* | Partial | Yes |
| DFUZB | Tarning-2012[14] | 380 | Africa | 2007—2007 | 7—600 | 42 | *msp-1, msp-2,*  *6 microsatellites* | Full | Non |
| DJYCQ | Zongo-2007a[15] | 261 | Africa | 2005—2005 | 6—564 | 28 | *msp-1, msp-2,*  *4 microsatellites* | Full | Yes |
| DTDDU | Faye-2007[16] | 525 | Africa | 2002—2003 | 12—900 | 28 | *msp-1* | Full | Yes |
| DYFKY | Awab-2010* | 59 | Asia | 2007—2010 | 24—624 | 56 | *msp-1, msp-2, glurp* | Full | Non |
| EDBXP | Karema-2006 [17] | 252 | Africa | 2003—2004 | 12—60 | 28 | *msp-1, msp-2* | Full | Yes |
| EDPJN | Abdulla-2008 [18] | 896 | Africa | 2006—2007 | 0—149 | 42 | *msp-1, msp-2, glurp* | Full | Yes |
| EGYMA | Sagara-2012 [19] | 680 | Africa | 2005—2005 | 6—560 | 28 | *msp-1, msp-2* | Full | Yes |
| EPDUY | Ashley-2004 [20] | 170 | Asia | 2003—2004 | 36—684 | 63 | *msp-1, msp-2, glurp* | Full | Yes |
| FEGFG | Valecha-2009 [21] | 124 | Asia | 2007—2007 | 24—660 | 28 | *msp-1, msp-2* | Full | Non |
| FEKFF | Agrawal-2013 [22] | 272 | Africa | 2010—2011 | 6—59 | 42 | *msp-1, msp-2, glurp* | Full | Yes |
| FFNAU | Grande-2007 [23] | 252 | S. America | 2003—2005 | 60—708 | 63 | *msp-1, msp-2, glurp* | Full | Yes |
| FSFKM | Kamugisha-2012 [24] | 108 | Africa | 2010—2010 | 4—60 | 28 | *msp-2* | Partial | Non |
| GARJK | Plucinski-2015 [25] | 399 | Africa | 2013—2013 | 6—108 | 28 | *7 microsatellites* | Partial | Non |
| GHNKU | Lefèvre-2001 [26] | 160 | Asia | 1998—1998 | 144—852 | 28 | *msp-1, msp-2, glurp* | Full | Yes |
| GPXJK | 4ABC Study -2011 [27] | 3,690 | Africa | 2007—2008 | 6—59 | 28 | *msp-1, msp-2, glurp* | Full | Yes |
| GTEFH | Van den Broek-2005 [28] | 121 | Asia | 2003—2003 | 30—792 | 42 | *msp-1, msp-2, glurp* | Full | Yes |
| GZQDA | Ngasala-2011a [29] | 359 | Africa | 2007—2008 | 4—60 | 56 | *msp-1, msp-2, glurp* | Unsupervised | Yes |
| HCEMT | Carrasquilla-2012 [30] | 159 | S. America | 2007—2008 | 144—672 | 42 | *Not stated* | Full | Yes |
| HJNDX | Temu-2010* | 102 | Africa | 2010—2011 | 7—58 | 28 | *msp-1, msp-2, glurp* | Full | Non |
| HKNHR | Mayxay-2004 [31] | 108 | Asia | 2002—2002 | 12—960 | 42 | *msp-1, msp-2, glurp* | Full | Yes |
| HMPBZ | Adam-2010[32] | 71 | Africa | 2009—2009 | 12—660 | 28 | *msp-2* | Full | Yes |
| JFPER | Djallé-2014 [33] | 128 | Africa | 2008—2009 | 2—59 | 28 | *msp-1, msp-2, glurp* | Full | Yes |
| JGGNM | Bassat-2009 [34] | 1,320 | Africa | 2005—2006 | 6—60 | 42 | *msp-1, msp-2, glurp* | Full | Yes |
| JTXEY | Achan-2009 [35] | 86 | Africa | 2007—2008 | 6—66 | 28 | *msp-1, msp-2,*  *4 microsatellites* | Partial | Yes |
| JXZNZ | Nikiema-2010* | 1,073 | Africa | 2000—2010 | 0—780 | 28 | *msp-1, msp-2* | Partial | Yes |
| KJGJT | Sirima-2009 [36] | 439 | Africa | 2004—2006 | 5—60 | 28 | *msp-1, msp-2, glurp* | Full | Yes |
| KPSGN | Haque-2007 [37] | 67 | Asia | 2005—2005 | 216—660 | 42 | *msp-1, glurp* | Full | Non |
| KRBXE | Smithuis-2006 [38] | 319 | Asia | 2003—2004 | 12—504 | 42 | *msp-1, msp-2, glurp* | Unsupervised | Yes |
| KZBZT | Mens-2008 [39] | 146 | Africa | 2007—2007 | 7—149 | 28 | *msp-1, msp-2, glurp* | Full | Yes |
| MCZHT | Yeka-2008 [40] | 414 | Africa | 2006—2007 | 6—120 | 42 | *msp-1, msp-2,*  *4 microsatellites* | Full | Yes |
| MEFSC | Premji-2009 [41] | 453 | Africa | 2006—2007 | 12—156 | 42 | *msp-1, msp-2, glurp* | Full | Yes |
| MPYTS | Mayxay-2012 [42] | 549 | Asia | 2010—2010 | 8—876 | 42 | *msp-1, msp-2, glurp* | Full | Yes |
| MTZZP | Mayxay-2006[43] | 107 | Asia | 2004—2004 | 16—600 | 42 | *msp-1, msp-2, glurp* | Full | Yes |
| NBSAE | Sagara-2008 [44] | 235 | Africa | 2004—2004 | 12—396 | 28 | *msp-1, msp-2,*  *1 microsatellite* | Full | Yes |
| PCPRC | Gadalla-2011 [45] | 86 | Africa | 2006—2006 | 18—792 | 28 | *msp-1, msp-2* | Partial | Non |
| PKGFP | van den Broek-2006 [46] | 106 | Africa | 2004—2004 | 12—59 | 28 | *msp-1, msp-2, glurp* | Full | Yes |
| PUEKP | Juma-2008 [40] | 267 | Africa | 2007—2007 | 6—62 | 28 | *msp-1, msp-2* | Full | Yes |
| QRBRC | Espié-2012 [47] | 299 | Africa | 2008—2009 | 6—59 | 42 | *msp-1, msp-2, glurp* | Full | Yes |
| QXJGK | Valecha-2010 [48] | 765 | Asia | 2005—2007 | 8—739 | 63 | *msp-1, msp-2, glurp* | Full | Yes |
| QZJGM | Borrmann-2011 [49] | 461 | Africa | 2005—2005 | 6—59 | 63 | *msp-1, msp-2, glurp* | Full | Yes |
| QZMAG | Sutanto-2013 [50] | 180 | Asia | 2008—2010 | 48—720 | 42 | *msp-1, msp-2, glurp* | Full | Yes |
| RDBXS | Grivoyannis-2009* | 334 | Africa | 2009—2009 | 12—732 | 28 | *msp-1, msp-2* | Partial | Non |
| REAJS | Ndiaye-2011 [51] | 354 | Africa | 2007—2008 | 10—780 | 28 | *msp-1, msp-2* | Full | Yes |
| RMRNH | Sirima-2015*[52]† | 927 | Africa | 2010—2013 | 6—60 | 63 | *msp-1, msp-2, glurp* | Full | Yes |
| RSBPS | Toure-2009 [53] | 60 | Africa | 2006—2006 | 18—600 | 28 | *msp-1, msp-2* | Partial | Yes |
| SATNJ | Offianan-2011 [54] | 242 | Africa | 2008—2008 | 6—59 | 28 | *msp-1, msp-2* | Full | Yes |
| SBCEE | Yeka-2014 [55] | 412 | Africa | 2008—2008 | 0—65 | 42 | *msp-1, msp-2, glurp* | Full | Yes |
| SFBSG | Carrara-2009 [56] | 197 | Asia | 2001—2002 | 36—840 | 63 | *msp-1, msp-2, glurp* | Full | Yes |
| SUEGP | Zongo-2007b [57] | 375 | Africa | 2006—2006 | 6—636 | 42 | *msp-1, msp-2,*  *4 microsatellites* | Full | Yes |
| SXGQP | Ursing-2011 [58] | 187 | Africa | 2006—2008 | 4—175 | 42 | *msp-1, msp-2, glurp* | Full | Yes |
| SYFQT | Martensson-2007 [59] | 50 | Africa | 2004—2004 | 6—135 | 42 | *msp-1, msp-2* | Partial | Yes |
| SZRDK | Ashley-2006 [60] | 196 | Asia | 2004—2005 | 10—780 | 63 | *msp-1, msp-2, glurp* | Full | Yes |
| TDFKY | Kamya-2007 [61] | 421 | Africa | 2006—2006 | 6—108 | 42 | *msp-1, msp-2,*  *4 microsatellites* | Full | Yes |
| TRKFC | Laminou-2008* | 192 | Africa | 2008—2008 | 5—192 | 28 | *Not stated* | Full | Non |
| TYKSC | Ndiaye-2009 [62] | 940 | Africa | 2006—2006 | 11—780 | 28 | *msp-1, msp-2,*  *1 microsatellite* | Full | Yes |
| UANQM | Karunajeewa-2008 [63] | 170 | Asia | 2005—2007 | 7—62 | 42 | *msp-1, msp-2, glurp* | Partial | Yes |
| UBTXH | Schramm-2013 [64] | 293 | Africa | 2008—2009 | 9—69 | 42 | *msp-1, msp-2, glurp* | Full | Yes |
| UGPAG | Bousema-2006 [65] | 75 | Africa | 2004—2004 | 6—120 | 28 | *msp-2* | Full | Yes |
| XEDNN | Yavo-2011 [66] | 384 | Africa | 2006—2007 | 24—924 | 28 | *msp-1, msp-2* | Full | Yes |
| XEKED | Faye-2010 [67] | 318 | Africa | 2007—2008 | 9—96 | 28 | *msp-1, msp-2* | Full | Yes |
| XTGNB | Hwang-2011 [68] | 112 | Africa | 2009—2009 | 9—720 | 42 | *msp-2* | Partial | Non |
| XXFCZ | Ngasala-2011b [69] | 244 | Africa | 2007—2007 | 5—61 | 42 | *msp-1, msp-2, glurp* | Unsupervised | Non |
| YGNQS | Sylla-2013 [70] | 393 | Africa | 2010—2010 | 24—756 | 42 | *msp-1, msp-2* | Full | Yes |
| YGTAH | Bukirwa-2006 [71] | 204 | Africa | 2004—2005 | 12—108 | 28 | *msp-1, msp-2* | Full | Yes |
| YPRHD | Price-2006 [72] | 419 | Asia | 2000—2001 | 24—792 | 42 | *msp-1, msp-2, glurp* | Full | Yes |
| YYDSM | Faucher-2009 [73] | 185 | Africa | 2007—2007 | 6—70 | 42 | *msp-1, msp-2* | Unsupervised | Yes |
| ZHKTN | Jones-2007* | 111 | Africa | 2007—2007 | 1—58 | 28 | *No parasitic recurrence* | Full | Yes |
| ZMNBX | Hien-2006* | 1,943 | Asia | 2002—2004 | 24—936 | 56 | *msp-1, msp-2, glurp* | Partial | Yes |
| ZYBXE | Martensson-2005 [74] | 200 | Africa | 2002—2003 | 6—60 | 42 | *msp-2* | Full | Yes |

* = Unpublished studies; *msp*-1 = meroziote surface protein-1; *msp*-2 = meroziote surface protein-2; *glurp* = glutamate rich protein

†Was unpublished at the time of data acquisition

## Estimation of hazard function of *P. falciparum* recrudescence

**Unadjusted hazard function**

The unadjusted hazard function was derived using the Kaplan-Meier method [75]. This assumes the hazard to be constant between successive failure times, and hence the hazard per unit time can be computed by dividing the width of the time interval ($\tau_{j})$. Let $d_{j}$ be the number of failures occurring within a time interval $\tau_{j}$ and $n_{j}$ be the number of people at risk at time *j*, the Kaplan-Meier type hazard is given by Collet (2015) (See equation 2.13 in p.32 of [75]):

$$h_{KM}=\frac{d_{j}}{n_{j}\tau_{j}}$$

The estimate of K-M type hazard is noisy and difficult to discern visible trends. Hence, smoothed estimates of the hazard function using kernel smoothing were also computed.

**Adjusted hazard function**

Parametric models and flexible parametric models, which use restricted cubic splines to model the hazard function (Royston-Parmar models) [76], were also considered for estimating the adjusted hazard function derived from regression models adjusting for mg/kg drug dosage and baseline parasitaemia.

**2.1 Kaplan-Meier type estimate of unadjusted hazard function of *P. falciparum* recrudescence**


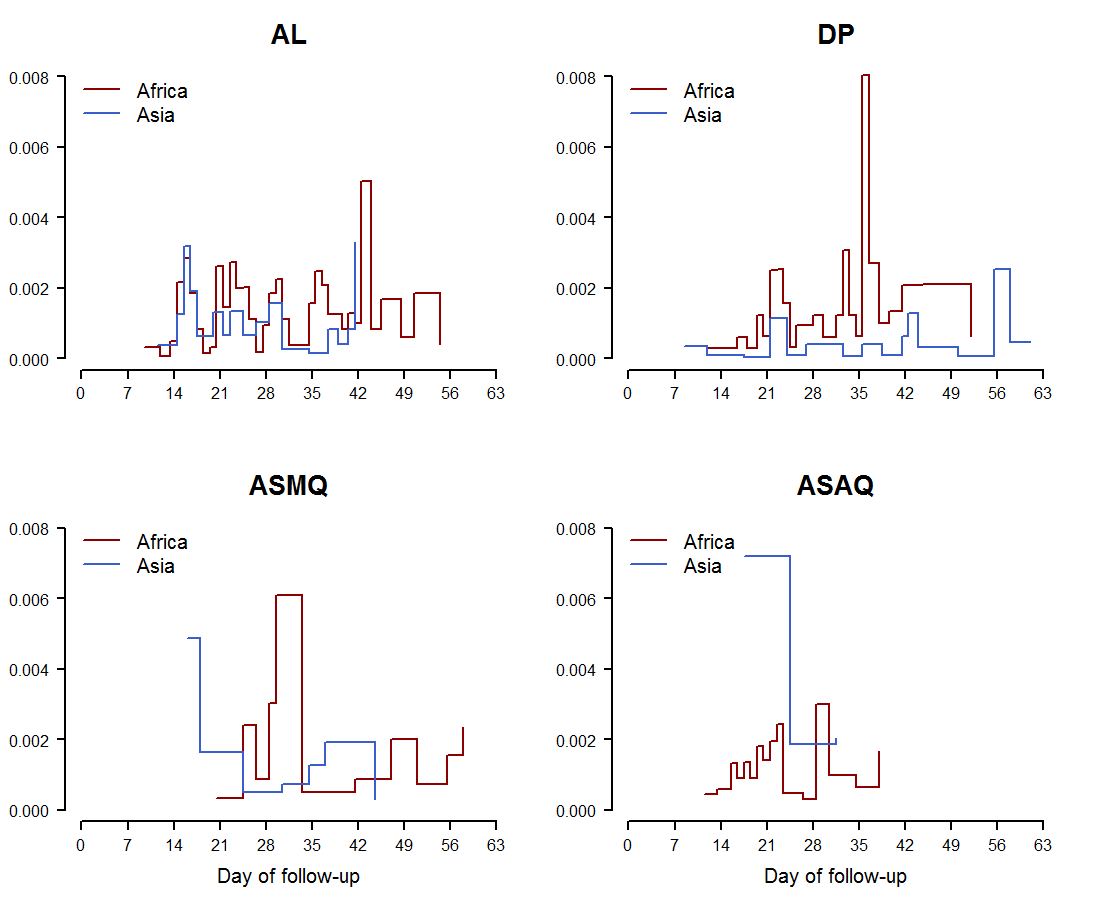


**Figure S2:**

**Legend**: The hazard function was estimated using **kphaz.fit** function in **muhaz** package in R software. AL = artemether-lumefantrine; DP = dihydroartemisinin-piperaquine; ASMQ = artesunate-mefloquine; ASAQ = artesunate-amodiaquine, and n = number of recrudescences. Only data from studies with at least three molecular markers are shown in the graph.

**2.2 Kernel-smoothed estimate of unadjusted hazard function of *P. falciparum* recrudescence**


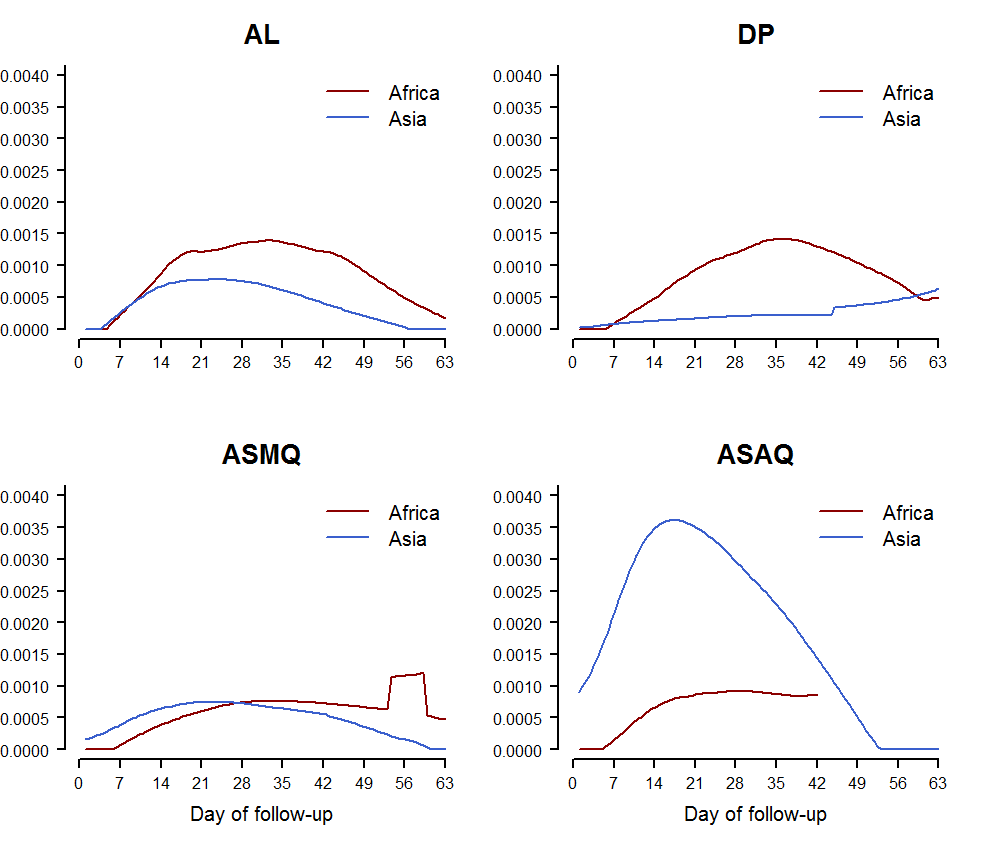


**Figure S3:**

**Legend**: The hazard function was estimated using muhaz function in **muhaz** package in R software using epacnechnikov kernel. AL = artemether-lumefantrine; DP = dihydroartemisinin-piperaquine; ASMQ = artesunate-mefloquine; ASAQ = artesunate-amodiaquine, and n = number of recrudescences. Only data from studies with at least three molecular markers are shown in the graph.

## Parametric survival analysis approach for estimating adjusted hazard function of *P. falciparum* recrudescence

**2.3.1** Estimates of adjusted hazard function **of *P. falciparum* recrudescence in children <5 years treated with artemether-lumefantrine (AL) in Africa**

| **** | **** |
| --- | --- |
| **** | **** |

**Figure S4:**

**Legend**: **Top panel**: The hazard function estimated using log-logistic regression and log-normal regression using **stata streg** command. These models adjusted for age, baseline parasitaemia and mg/kg drug dose of partner component. **Bottom panel**: Assessment of goodness of fit of the parametric models using Cox-Snell’s residuals. The residuals should fall along the 45 degrees line for a good-fitting model.

**2.3.2 Estimates of adjusted hazard function of *P. falciparum* recrudescence in patient of all ages treated with artemether-lumefantrine (AL) in Asia**

| **** | **** |
| --- | --- |
| **** | **** |

**Figure S5:**

**Legend**: **Top panel**: The hazard function estimated using log-logistic regression and log-normal regression using **stata streg** command. These models adjusted for age, baseline parasitaemia and mg/kg drug dose of partner component. **Bottom panel**: Assessment of goodness of fit of the parametric models using Cox-Snell’s residuals. The residuals should fall along the 45 degrees line for a good-fitting model.

**2.3.3 Estimates of adjusted hazard function of *P. falciparum* recrudescence in children <5 years treated with dihydroartemisinin-piperaquine (DP) in Africa**

| **** | **** |
| --- | --- |
| **** | **** |

**Figure S6:**

**Legend**: **Top panel**: The hazard function estimated using log-logistic regression and log-normal regression using **stata streg** command. These models adjusted for age, baseline parasitaemia and mg/kg drug dose of partner component. **Bottom panel**: Assessment of goodness of fit of the parametric models using Cox-Snell’s residuals. The residuals should fall along the 45 degrees line for a good-fitting model.

**2.3.4 Flexible parametric (Royston-Parmar model) approach for estimating the adjusted hazard of *P. falciparum* recrudescence in patients treated with artemether-lumefantrine and dihydroartemisinin-piperaquine**

In Africa, the analysis was carried out in children < 5 years for artemether-lumefantrine and dihydroartemisinin-piperaquine. For AL in Asia, patient of all ages were included. The AIC estimates for Royston-Parmar models with different number of knots are shown below:

**Table S2: Akaika’s Information Criteria (AIC) for Royston-Parmar model**

| Knots | AL Africa† | AL Asia | DP Africa |
| --- | --- | --- | --- |
| *k*=0 | 2759.1 | 647.2 | 1319.8 |
| *k*=1 | 2707.9 | 630.6 | 1305.9 |
| *k*=2 | 2707.7 | 631.4 | 1307.5 |
| *k*=3 | 2707.4 | 633.6 | 1306.6 |

†For AL Africa, *k*=1 chosen as the model of choice for parsimonious reasons. The Royston-Parmar model for AL Africa was adjusted for age, baseline parasitaemia and mg/kg drug dosage with study sites fitted as shared frailty term. For AL Asia, the model adjusted for age and baseline parasitaemia. For DP Africa, the model adjusted for age, parasitaemia and mg/kg dose.

The hazard functions corresponding to knots = 1,2, and 3 presented in Table 4A.1 are plotted below:


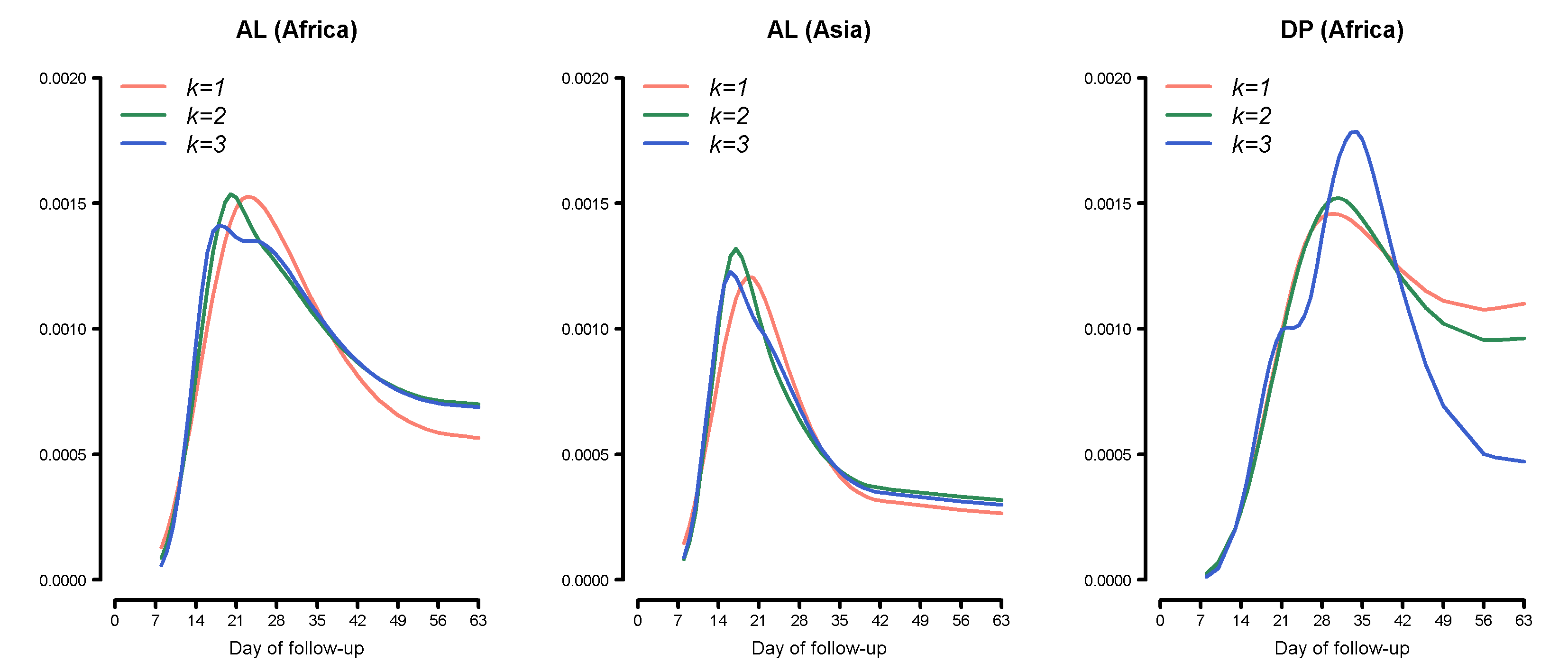


**Figure S7:**

AL = artemether-lumefantrine; DP = dihydroartemisinin-piperaquine

## Using multivariable fractional polynomials to estimate hazard function of *P. falciparum* recrudescence

Parametric models, which can accommodate non-monotone hazards (log-normal and log-logistic) were found to be poor fitting based on Cox-Snell’s residuals (Section 2). The hazard functions derived from Royston-Parmar models, which utilises restricted cubic splines, were of similar shape to the ones obtained from fractional polynomial smoothing. Since splines are mathematically complicated equations and difficult to utilise for simulation purposes, they weren’t considered any further and the rest of the section outlines the statistical approach of estimating the hazard function using fractional polynomials.

The algorithm for estimation of hazard function is outlined below:

Let,

$$\ln\left\{ H_{0}\left( t \right) \right)=\boldsymbol{\alpha}\times f\left( \ln\left( time \right) \right)=z_{0}(t)$$

where $\boldsymbol{\alpha}$ is a vector of coefficients for different fractional polynomial transformations. Using the relationship between baseline hazard and cumulative baseline hazard:

$$h_{0}\left( t \right)=\frac{dH_{0}(t)}{dt}$$

$$\Rightarrow h_{0}\left( t \right)=\frac{dH_{0}(t)}{dt}\times\frac{d ln(H_{0}\left( t \right))}{d ln(t)}\times\frac{d ln(t)}{d ln(H_{0}\left( t \right))}$$

$$\Rightarrow h_{0}\left( t \right)=\frac{d ln(t)}{dt}\times\frac{d ln(H_{0}\left( t \right))}{d ln(t)}\times\frac{dH_{0}(t)}{d ln(H_{0}\left( t \right))}$$

Differentiating each term of the expression:

$$\frac{d\ln\left( t \right)}{dt}=\frac{1}{t}$$

$$\frac{d\ln\left( H_{0}\left( t \right) \right)}{d\ln\left( t \right)}=\frac{dz_{0}\left( t \right)}{d\ln\left( t \right)}$$

$$\frac{dH_{0}\left( t \right)}{d\ln\left( H_{0}\left( t \right) \right)}=\frac{1}{\left( \frac{d\ln\left( H_{0}\left( t \right) \right)}{dH_{0}\left( t \right)} \right)}=\frac{1}{\frac{1}{H_{0}(t)}}=H_{0}\left( t \right)=exp(z_{0}\left( t \right))$$

This gives an estimate of the smoothed baseline hazard:

$$h_{0}\left( t \right)=\frac{1}{t}\times\frac{{dz}_{0}\left( t \right)}{d\ln\left( t \right)}\times\exp\left( z_{0}\left( t \right) \right)$$

## Estimated equations of hazard function using fractional polynomial smoothing method

Based on the algorithm outlined above, the following equations were obtained for cumulative baseline hazard for recrudescence for AL and DP in Africa and Asia.

**AL (Africa):**

$$\ln\left( CBH \right)= -3.680424+10.49305\times\left\{ ln(t)-3.427413427 \right\}-1.258464\times\left\{ {\ln\left( t \right)}^{2}-11.7471628 \right\}$$

**AL (Asia):**

$$\ln\left( CBH \right)= -3.955812+2.812377\times\left\{ {ln(t)}^{2}-12.29012325 \right\}-1.521957\times\left\{ {\ln\left( t \right)}^{2}\times ln(\ln\left( t \right))-15.41670573 \right\}$$

**DP (Africa overall):**

$$\ln\left( CBH \right)=-4.339292+0.8416194\times\left\{ {ln(t)}^{3}-37.4570925 \right\}-0.4869875\times\left\{ {\ln\left( t \right)}^{3}\times ln(\ln\left( t \right))-45.23813021 \right\}$$

**DP (Africa; (<48 mg/kg of piperaquine):**

$$\ln\left( CBH \right)=-3.162359 +0.9712495\times\left\{ {ln(t)}^{3}-47.21631182 \right\}-0.5727575\times\left\{ {\ln\left( t \right)}^{3}\times ln(\ln\left( t \right))-60.66885952 \right\}$$

**DP (Africa; (**≥**48 mg/kg of piperaquine):**

$$\ln\left( CBH \right)=-4.021096 +0.5752936\times\left\{ {ln(t)}^{3}-43.50176377 \right\}-0.3197093\times\left\{ {\ln\left( t \right)}^{3}\times ln(\ln\left( t \right))-54.70783962 \right\}$$

CBH = Cumulative baseline hazard

## Simulation studies: design and objectives

This simulation study had two aims:

1. To quantify the absolute overestimation in the efficacy estimates for AL and DP with a day 28 and day 42 follow-up duration, respectively.
2. To investigate the impact of duration of follow-up on the relative risk estimates for drug regimens with different half-lives (using AL and DP as motivating example).

The followings steps define the simulation set-up:

## 4.1 Simulation set-up

1. Time to recrudescence $\left( t_{rc} \right)$was generated using the following cumulative baseline hazard (CBH) presented in Section 3.1:

$\ln\left( CBH\left( t \right)_{rc} \right)= -3.680424+10.49305\times\left\{ ln(t)-3.427413427 \right\}-1.258464\times\left\{ {\ln\left( t \right)}^{2}-11.7471628 \right\}$ for artemether-lumefantrine

$\ln\left( CBH \left( t \right)_{rc} \right)=-4.021096 +0.5752936\times\left\{ {ln(t)}^{3}-43.50176377 \right\}-0.3197093\times\left\{ {\ln\left( t \right)}^{3}\times ln(\ln\left( t \right))-54.70783962 \right\}$ for dihydroartemisinin-piperaquine

These functions approximately assigned 4% of the observations to be recrudescence for both AL and DP regimen.

1. Simulate time to new infections $\left( t_{ni} \right)$ using:

$\ln\left( CB{H(t)}_{ni} \right)=\alpha_{0}+ 9501.2150\times\left\{ {\ln\left( t \right)}^{-2}-0.0858 \right\}-31651.33\times\{{\ln\left( t \right)}^{-2}\times\ln\left( \ln\left( t \right)-0.1054 \right\} +29340.83\times\left\{ {lnt}^{-2}\times{\ln\left( lnt \right)}^{2}-0.1294 \right\}-12690.51\times\{{\ln\left( t \right)}^{-2}\times{\ln\left( \ln\left( t \right) \right)}^{3}-0.1588\}$ for artemether-lumefantrine

The parameter $\alpha_{0}$ was varied to achieve the desired proportion of new infections

$\alpha_{0}=-4.190984$ for approximately 15% new infections

$\alpha_{0}=-3.290984$ for approximately 30% new infections

$\ln\left( CB{H(t)}_{ni} \right)=\beta_{0}+ 9501.2150\times\left\{ {\ln\left( t \right)}^{-2}-0.0858 \right\}-31651.33\times\{{\ln\left( t \right)}^{-2}\times\ln\left( \ln\left( t \right)-0.1054 \right\} +29340.83\times\left\{ {lnt}^{-2}\times{\ln\left( lnt \right)}^{2}-0.1294 \right\}-12690.51\times\left\{ {\ln\left( t \right)}^{-2}\times{\ln\left( \ln\left( t \right) \right)}^{3}-0.1588 \right\}$ for dihydroartemisinin-piperaquine

The parameter $\beta_{0}$ was varied to achieve the desired proportion of new infections

$\beta_{0}=-4.800422$ for approximately <10% new infections

$\beta_{0}=-4.190422$ for approximately 15% new infections

The shape of the survival and hazard functions used for the simulation study is presented in Figure 8.

1. Since early recurrences are unlikely, the minimum time was set to day 14 and administrative censoring was applied on the last scheduled follow-up visit (day 63). For simplicity, no losses to follow-up were assumed.
2. For each individual, the observed time $(t)$ was defined as the minimum of the simulated time to recrudescence and new infection. The observed event corresponded to the event with minimum time*.* The final observed time was rounded to the nearest weekly visit day (7, 14, 21 and so on), reflective of the antimalarial follow-up design.
3. Kaplan-Meier estimates for AL and DP were computed on days 28, 42 and 63 and stored. Cox’s regression model was fitted and the estimate of hazards ratio (HR) for AL relative to DP on days 28, 35, 42, 56, and 63 were stored.
4. This was repeated 1,000 times and the median (interquartile range) of the estimated HRs were then reported for a simulation study with following sample sizes: n=200, 500, and 1,000 subjects/arm.


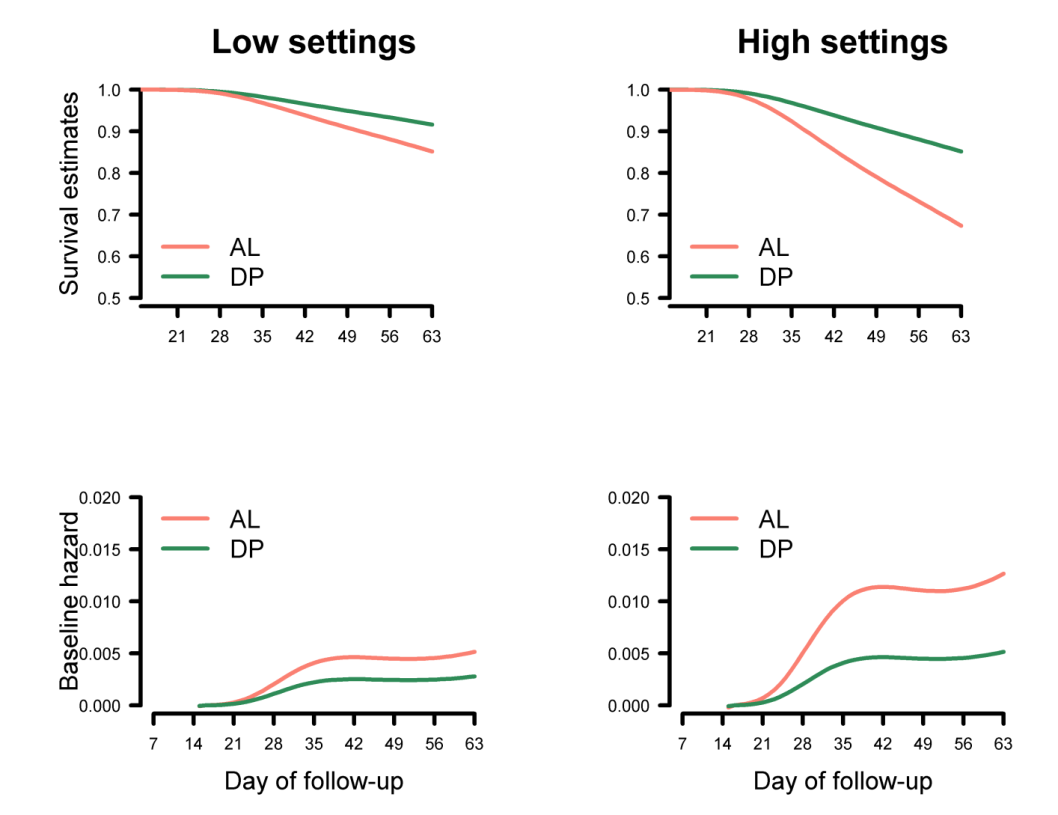


**Figure S8:** The survival and hazard function used for the simulation study for the comparative efficacy is given below. The hazard ratio derived from Cox regression model is presented in the table below.

AL = artemether-lumefantrine; DP = dihydroartemisinin-piperaquine

**4.2 Additional results from the simulation study designed for comparing two drug regimens**

Table: Estimated hazard ratio for artemether-lumefantrine relative to dihydroartemisinin-piperaquine at different follow-up time point

**Table S3:**

|  | **Low Settings** | | | **High Settings** | | |
| --- | --- | --- | --- | --- | --- | --- |
| **Day** | n=200 subjects | n=500 subjects | n=1,000 subjects | n=200 subjects | n=500 subjects | n=1,000 subjects |
| 28 | 2.01 [1.00 - 4.04] | 2.00 [1.29 - 2.78] | 1.89 [1.46 - 2.51] | 2.01 [1.01 - 4.04] | 1.85 [1.34 - 3.02] | 1.90 [1.45 - 2.53] |
| 35 | 1.62 [1.01 - 2.69] | 1.57 [1.19 - 2.03] | 1.57 [1.27 - 1.91] | 1.67 [1.01 - 2.74] | 1.59 [1.18 - 2.21] | 1.58 [1.28 - 1.94] |
| 42 | 1.35 [0.89 - 2.04] | 1.30 [1.07 - 1.68] | 1.33 [1.12 - 1.59] | 1.39 [0.91 - 2.06] | 1.36 [1.05 - 1.76] | 1.35 [1.11 - 1.63] |
| 49 | 1.16 [0.83 - 1.67] | 1.15 [0.95 - 1.43] | 1.16 [1.01 - 1.38] | 1.22 [0.85 - 1.72] | 1.17 [0.96 - 1.48] | 1.19 [1.00 - 1.39] |
| 56 | 1.03 [0.76 - 1.44] | 1.05 [0.87 - 1.29] | 1.05 [0.92 - 1.23] | 1.08 [0.77 - 1.5] | 1.07 [0.89 - 1.30] | 1.08 [0.93 - 1.24] |
| 63 | 0.94 [0.71 - 1.32] | 0.98 [0.81 - 1.19] | 0.97 [0.84 - 1.12] | 1.01 [0.73 - 1.39] | 0.99 [0.82 - 1.21] | 1.00 [0.87 - 1.15] |

AL = artemether-lumefantrine; DP = dihydroartemisinin-piperaquine

Low settings = approximately 8% new infections with AL and 15% new infections in DP arm

High settings = approximately 15% new infections with AL and 30% new infections in DP arm

## Sensitivity analyses

## The estimated area under the curve (AUC) of constructed distribution for *P. falciparum* recrudescence using different “observed time of recrudescence”

In Africa, the analysis was carried out in children < 5 years for artemether-lumefantrine and dihydroartemisinin-piperaquine. For AL in Asia, patient of all ages were included.

**Table S4: The AUC of the probability distribution from day 42 studies (presented in main text)**

|  | **Day 42 studies** | | | |
| --- | --- | --- | --- | --- |
| Day | AL Africa | AL Asia | DP (UD) | DP (nUD) |
| 28 | 0.55 | 0.70 | 0.37 | 0.35 |
| 35 | 0.80 | 0.90 | 0.67 | 0.59 |
| 42 | 0.95 | 0.99 | 0.90 | 0.80 |
| 49 | 1.00 | 1.00 | 0.99 | 0.94 |
| 56 | 1.00 | 1.00 | 1.00 | 1.00 |
| 63 | 1.00 | 1.00 | 1.00 | 1.00 |

AL = artemether-lumefantrine; DP = dihydroartemisinin-piperaquine; UD = Under-dosed (piperaquine dose ≤48 mg/kg); nUD= Not Under-dosed (piperaquine dose > 48 mg/kg)

**Table S5: The AUC of the probability distribution using different definition of observed time of P. falciparum recrudescence**

|  | **Assumption 1^a^** | | | | **Assumption 2^b^** | | | |
| --- | --- | --- | --- | --- | --- | --- | --- | --- |
| Day | AL Africa | AL Asia | DP (UD) |  | AL Africa | AL Asia | DP (UD) |  |
| 28 | 0.52 | 0.72 | 0.44 |  | 0.40 | 0.72 | 0.41 |  |
| 35 | 0.74 | 0.91 | 0.71 |  | 0.63 | 0.92 | 0.72 |  |
| 42 | 0.89 | 0.99 | 0.91 |  | 0.81 | 1.00 | 0.93 |  |
| 49 | 0.97 | 1.00 | 0.99 |  | 0.93 | 1.00 | 1.00 |  |
| 56 | 1.00 | 1.00 | 1.00 |  | 0.99 | 1.00 | 1.00 |  |
| 63 | 1.00 | 1.00 | 1.00 |  | 1.00 | 1.00 | 1.00 |  |

AL = artemether-lumefantrine; DP = dihydroartemisinin-piperaquine; UD = Under-dosed (piperaquine dose <= 48 mg/kg); nUD= Not Under-dosed (piperaquine dose > 48 mg/kg)

**^a^**Assuming recrudescence occurred the day after the last visit where patient recorded negative parasitaemia on blood smear; ^b^ Assuming recrudescence occurred half-way through the last visit with recorded negative smear and the day when it recorded in the study.

## Results from analysis with multiply imputed dataset on indeterminant PCR outcomes

Distribution of PCR confirmed recrudescence obtained from imputing indeterminant PCR outcomes. The red line is the average from 100 imputations.

AL in Africa children <5 years


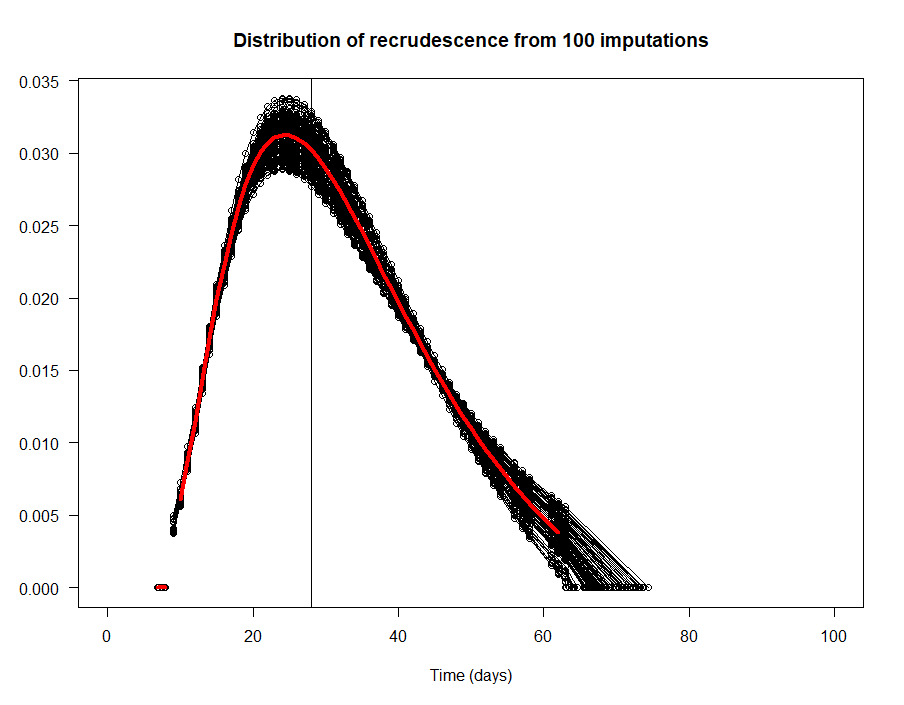


AL in patient of all ages in Asia or S America


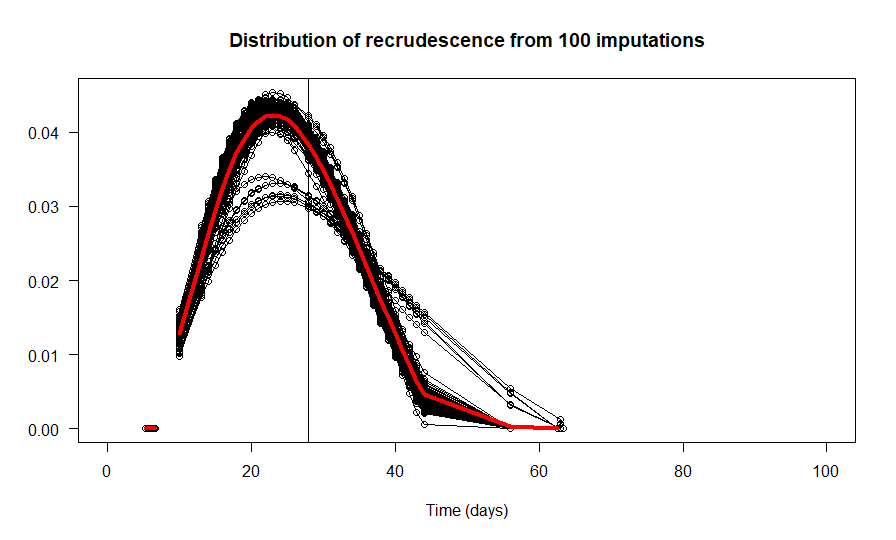


**Table S6: The area under the curve of the estimated probability distribution from 100 multiple imputations for AL in Asia and Africa**

|  | Africa | | Asia | | |
| --- | --- | --- | --- | --- | --- |
| Day | AL in Africa  [95% CI] | Multiple Imputations  (m=100) ^a^ | AL in Asia  [95% CI] | Multiple Imputations  (m=100) ^b^ |  |
| 28 | 0.42 [0.26 – 0.53] | 0.44 | 0.68 [0.55 - 0.85] | 0.64 |  |
| 35 | 0.63 [0.40 – 0.78] | 0.64 | 0.88 [0.78 - 0.99] | 0.86 |  |
| 42 | 0.79 [0.53 – 0.97] | 0.79 | 0.98 [0.93 - 1.00] | 0.98 |  |
| 49 | 0.91 [0.64 – 1.00] | 0.89 | 1.00 [0.93 - 1.00] | 0.99 |  |
| 56 | 0.97 [0.74 – 1.00] | 0.95 | 1.00 [0.94 - 1.00] | 0.99 |  |
| 63 | 1.00 [0.81 – 1.00] | 0.99 | 1.00 | 1 |  |

^a^ 302 recurrences with missing PCR outcomes in Africa

^b^ 79 indeterminant outcomes in Asia

## References

1. The WorldWide Antimalarial Resistance Network (WWARN) AS-AQ Study Group. The effect of dosing strategies on the therapeutic efficacy of artesunate-amodiaquine for uncomplicated malaria: a meta-analysis of individual patient data. BMC Med. 2015;13:66.

2. Bouyou-Akotet MK, Ramharter M, Ngoungou EB, Mamfoumbi MM, Mihindou MP, Missinou MA, et al. Efficacy and safety of a new pediatric artesunatemefloquine drug formulation for the treatment of uncomplicated falciparum malaria in Gabon. Wien Klin Wochenschr. 2010;122:173–8.

3. Menan H, Faye O, Same-Ekobo A, Oga ASS, Faye B, Kiki Barro CP, et al. Comparative study of the efficacy and tolerability of dihydroartemisinin - piperaquine - trimethoprim versus artemether - lumefantrine in the treatment of uncomplicated Plasmodium falciparum malaria in Cameroon, Ivory Coast and Senegal. Malar J. 2011;10:185.

4. Mutabingwa TK, Anthony D, Heller A, Hallett R, Ahmed J, Drakeley C, et al. Amodiaquine alone, amodiaquine+sulfadoxine-pyrimethamine, amodiaquine+artesunate, and artemether-lumefantrine for outpatient treatment of malaria in Tanzanian children: A four-arm randomised effectiveness trial. Lancet. 2005;365:1474–80.

5. Hien TT, Thuy-Nhien NT, Phu NH, Boni MF, Thanh NV, Nha-Ca NT, et al. In vivo susceptibility of plasmodium falciparum to artesunate in Binh Phuoc Province, Vietnam. Malar J. Malaria Journal; 2012;11:355.

6. Piola P, Fogg C, Bajunirwe F, Biraro S, Grandesso F, Ruzagira E, et al. Supervised versus unsupervised intake of six-dose artemether-lumefantrine for treatment of acute, uncomplicated Plasmodium falciparum malaria in Mbarara, Uganda: A randomised trial. Lancet. 2005;365:1467–73.

7. Smithuis F, Kyaw MK, Phe O, Win T, Aung PP, Oo APP, et al. Effectiveness of five artemisinin combination regimens with or without primaquine in uncomplicated falciparum malaria: An open-label randomised trial. Lancet Infect Dis. 2010;10:673–81.

8. Thanh NX, Trung TN, Phong NC, Thien NX, Dai B, Shanks GD, et al. Open label randomized comparison of dihydroartemisinin-piperaquine and artesunate-amodiaquine for the treatment of uncomplicated Plasmodium falciparum malaria in central Vietnam. Trop Med Int Heal. 2009;14:504–11.

9. Janssens B, van Herp M, Goubert L, Chan S, Uong S, Nong S, et al. A randomized open study to assess the efficacy and tolerability of dihydroartemisinin-piperaquine for the treatment of uncomplicated falciparum malaria in Cambodia. Trop Med Int Heal. 2007;12:251–9.

10. Falade C, Makanga M, Premji Z, Ortmann CE, Stockmeyer M, Ibarra de Palacios P. Efficacy and safety of artemether-lumefantrine (Coartem®) tablets (six-dose regimen) in African infants and children with acute, uncomplicated falciparum malaria. Trans R Soc Trop Med Hyg. 2005;99:459–67.

11. Arinaitwe E, Sandison TG, Wanzira H, Kakuru A, Homsy J, Kalamya J, et al. Artemether-Lumefantrine versus Dihydroartemisinin- Piperaquine for Falciparum Malaria: A Longitudinal, Randomized Trial in Young Ugandan Children. Clin Infect Dis. 2009;49:1629–37.

12. Sawa P, Shekalaghe SA, Drakeley CJ, Sutherland CJ, Mweresa CK, Baidjoe AY, et al. Malaria transmission after artemether-lumefantrine and dihydroartemisinin- piperaquine: A randomized trial. J Infect Dis. 2013;207:1637–45.

13. Clark TD, Njama-Meya D, Nzarubara B, Maiteki-Sebuguzi C, Greenhouse B, Staedke SG, et al. Incidence of malaria and efficacy of combination antimalarial therapies over 4 years in an urban cohort of Ugandan children. PLoS One. 2010;5:1–8.

14. Tarning J, Zongo I, Somé F a, Rouamba N, Parikh S, Rosenthal PJ, et al. Population Pharmacokinetics and Pharmacodynamics of Piperaquine in Children With Uncomplicated Falciparum Malaria. Clin Pharmacol Ther. Nature Publishing Group; 2012;91:497–505.

15. Zongo I, Dorsey G, Rouamba N, Tinto H, Dokomajilar C, Guiguemde RT, et al. Artemether-lumefantrine versus amodiaquine plus sulfadoxine-pyrimethamine for uncomplicated falciparum malaria in Burkina Faso: a randomised non-inferiority trial. Lancet. 2007;369:491–8.

16. Faye B, Ndiaye J-L, Ndiaye D, Dieng Y, Faye O, Gaye O. Efficacy and tolerability of four antimalarial combinations in the treatment of uncomplicated Plasmodium falciparum malaria in Senegal. Malar J. 2007;6:80.

17. Karema C, Fanello CI, Van Overmeir C, Van geertruyden JP, van Doren W, Ngamije D, et al. Safety and efficacy of dihydroartemisinin/piperaquine (Artekin®) for the treatment of uncomplicated Plasmodium falciparum malaria in Rwandan children. Trans R Soc Trop Med Hyg. 2006;100:1105–11.

18. Abdulla S, Sagara I, Borrmann S, D’Alessandro U, Gonzalez R, Hamel M, et al. Efficacy and safety of artemether-lumefantrine dispersible tablets compared with crushed commercial tablets in African infants and children with uncomplicated malaria: a randomised, single-blind, multicentre trial. Lancet. 2008;372:1819–27.

19. Sagara I, Fofana B, Gaudart J, Sidibe B, Togo A, Toure S, et al. Repeated artemisinin-based combination therapies in a malaria hyperendemic area of Mali: efficacy, safety, and public health impact. Am J Trop Med Hyg. 2012;87:50–6.

20. Ashley EA, Krudsood S, Phaiphun L, Srivilairit S, McGready R, Leowattana W, et al. Randomized, controlled dose-optimization studies of dihydroartemisinin-piperaquine for the treatment of uncomplicated multidrug-resistant falciparum malaria in Thailand. J Infect Dis. 2004;190:1773–82.

21. Valecha N, Srivastava P, Mohanty SS, Mittra P, Sharma SK, Tyagi PK, et al. Therapeutic efficacy of artemether-lumefantrine in uncomplicated falciparum malaria in India. Malar J. 2009;8:107.

22. Agarwal A, McMorrow M, Onyango P, Otieno K, Odero C, Williamson J, et al. A randomized trial of artemether-lumefantrine and dihydroartemisinin-piperaquine in the treatment of uncomplicated malaria among children in western Kenya. Malar J. Malaria Journal; 2013;12:254.

23. Grande T, Bernasconi A, Erhart A, Gamboa D, Casapia M, Delgado C, et al. A randomised controlled trial to assess the efficacy of dihydroartemisinin-piperaquine for the treatment of uncomplicated falciparum malaria in Peru. PLoS One. 2007;2:e1101.

24. Kamugisha E, Jing S, Minde M, Kataraihya J, Kongola G, Kironde F, et al. Efficacy of artemether-lumefantrine in treatment of malaria among under-fives and prevalence of drug resistance markers in Igombe-Mwanza, north-western Tanzania. Malar J. BioMed Central Ltd; 2012;11:58.

25. Plucinski MM, Talundzic E, Morton L, Dimbu PR, Macaia AP, Fortes F, et al. Efficacy of artemether-lumefantrine and dihydroartemisinin-piperaquine for treatment of uncomplicated malaria in children in Zaire and Uige provinces, Angola. Antimicrob Agents Chemother. 2015;59:437–43.

26. Lefèvre G, Looareesuwan S, Treeprasertsuk S, Krudsood S, Silachamroon U, Gathmann I, et al. A clinical and pharmacokinetic trial of six doses of artemether-lumefantrine for multidrug-resistant Plasmodium falciparum malaria in Thailand. Am J Trop Med Hyg. 2001;64:247–56.

27. The Four Artemisinin-Based Combinations (4ABC) Study group. A head-to-head comparison of four artemisinin-based combinations for treating uncomplicated malaria in african children: A randomized trial. PLoS Med. 2011;8.

28. van den Broek I V, Maung UA, Peters A, Liem L, Kamal M, Rahman M, et al. Efficacy of chloroquine + sulfadoxine-pyrimethamine, mefloquine + artesunate and artemether + lumefantrine combination therapies to treat Plasmodium falciparum malaria in the Chittagong Hill Tracts, Bangladesh. Trans R Soc Trop Med Hyg. 2005;99:727–35.

29. Ngasala BE, Malmberg M, Carlsson AM, Ferreira PE, Petzold MG, Blessborn D, et al. Effectiveness of artemether-lumefantrine provided by community health workers in under-five children with uncomplicated malaria in rural Tanzania: An open label prospective study. Malar J. 2011;10:1–10.

30. Carrasquilla G, Barón C, Monsell EM, Cousin M, Walter V, Lefèvre G, et al. Randomized, prospective, three-arm study to confirm the auditory safety and efficacy of artemether-lumefantrine in Colombian patients with uncomplicated Plasmodium falciparum malaria. Am J Trop Med Hyg. 2012;86:75–83.

31. Mayxay M, Khanthavong M, Lindegardh N, Keola S, Barends M, Pongvongsa T, et al. Randomized Comparison of Chloroquine plus Sulfadoxine-Pyrimethamine versus Artesunate plus Mefloquine versus Artemether-Lumefantrine in the Treatment of Uncomplicated Falciparum Malaria in the Lao People’s Democratic Republic. Clin Infect Dis. 2004;39:1139–47.

32. Adam I, Salah MT, Eltahir HG, Elhassan a H, Elmardi K a, Malik EM. Dihydroartemisinin-piperaquine versus artemether-lumefantrine, in the treatment of uncomplicated Plasmodium falciparum malaria in central Sudan. Ann Trop Med Parasitol. 2010;104:319–26.

33. Djallé D, Njuimo SP, Manirakiza A, Laganier R, Le Faou A, Rogier C. Efficacy and safety of artemether + lumefantrine, artesunate + sulphamethoxypyrazine-pyrimethamine and artesunate + amodiaquine and sulphadoxine-pyrimethamine + amodiaquine in the treatment of uncomplicated falciparum malaria in Bangui, Central African Re. Malar J. 2014;13:1–9.

34. Bassat Q, Mulenga M, Tinto H, Piola P, Borrmann S, Menéndez C, et al. Dihydroartemisinin-piperaquine and artemether-lumefantrine for treating uncomplicated malaria in African children: A randomised, non-inferiority trial. PLoS One. 2009;4.

35. Achan J, Tibenderana JK, Kyabayinze D, Wabwire Mangen F, Kamya MR, Dorsey G, et al. Effectiveness of quinine versus artemether-lumefantrine for treating uncomplicated falciparum malaria in Ugandan children: randomised trial. Br Med J. 2009;339:b2763.

36. Sirima SB, Tiono AB, Gansané A, Diarra A, Ouédraogo A, Konaté AT, et al. The efficacy and safety of a new fixed-dose combination of amodiaquine and artesunate in young African children with acute uncomplicated Plasmodium falciparum. Malar J. 2009;8:48.

37. Haque R, Thriemer K, Wang Z, Sato K, Wagatsuma Y, Salam MA, et al. Therapeutic efficacy of artemether-lumefantrine for the treatment of uncomplicated Plasmodium falciparum malaria in Bangladesh. Am J Trop Med Hyg. 2007;76:39–41.

38. Smithuis F, Kyaw MK, Phe O, Aye KZ, Htet L, Barends M, et al. Efficacy and effectiveness of dihydroartemisinin-piperaquine versus artesunate-mefloquine in falciparum malaria : an open-label randomised comparison. Lacent. 2006;367:2075–85.

39. Mens PF, Sawa P, Van Amsterdam SM, Versteeg I, Omar SA, Schallig HDFH, et al. A randomized trial to monitor the efficacy and effectiveness by QT-NASBA of artemether-lumefantrine versus dihydroartemisinin-piperaquine for treatment and transmission control of uncomplicated Plasmodium falciparum malaria in western Kenya. Malar J. 2008;7:237.

40. Yeka A, Dorsey G, Kamya MR, Talisuna A, Lugemwa M, Rwakimari JB, et al. Artemether-lumefantrine versus dihydroartemisinin-piperaquine for treating uncomplicated malaria: a randomized trial to guide policy in Uganda. PLoS One. 2008;3:e2390.

41. Premji Z, Umeh RE, Owusu-Agyei S, Esamai F, Ezedinachi EU, Oguche S, et al. Chlorproguanil-dapsone-artesunate versus artemether-lumefantrine: a randomized, double-blind phase III trial in African children and adolescents with uncomplicated Plasmodium falciparum malaria. PLoS One. 2009;4:e6682.

42. Mayxay M, Khanthavong M, Chanthongthip O, Imwong M, Pongvongsa T, Hongvanthong B, et al. Efficacy of artemether-lumefantrine, the nationally-recommended artemisinin combination for the treatment of uncomplicated falciparum malaria, in southern Laos. Malar J. 2012;11:1–10.

43. Mayxay M, Thongpraseuth V, Khanthavong M, Lindegårdh N, Barends M, Keola S, et al. An open, randomized comparison of artesunate plus mefloquine vs. dihydroartemisinin-piperaquine for the treatment of uncomplicated Plasmodium falciparum malaria in the Lao People’s Democratic Republic (Laos). Trop Med Int Heal. 2006;11:1157–65.

44. Sagara I, Diallo A, Kone M, Coulibaly M, Diawara SI, Guindo O, et al. A randomized trial of artesunate-mefloquine versus artemether-lumefantrine for treatment of uncomplicated Plasmodium falciparum malaria in Mali. Am J Trop Med Hyg. 2008;79:655–61.

45. Gadalla NB, Adam I, Elzaki SE, Bashir S, Mukhtar I, Oguike M, et al. Increased pfmdr1 copy number and sequence polymorphisms in Plasmodium falciparum isolates from Sudanese malaria patients treated with artemether-lumefantrine. Antimicrob Agents Chemother. 2011;55:5408–11.

46. van den Broek I, Kitz C, Al Attas S, Libama F, Balasegaram M, Guthmann JP. Efficacy of three artemisinin combination therapies for the treatment of uncomplicated Plasmodium falciparum malaria in the Republic of Congo. Malar J. 2006;5:113.

47. Espié E, Lima A, Atua B, Dhorda M, Flévaud L, Sompwe EM, et al. Efficacy of fixed-dose combination artesunate-amodiaquine versus artemether-lumefantrine for uncomplicated childhood Plasmodium falciparum malaria in Democratic Republic of Congo: A randomized non-inferiority trial. Malar J. 2012;11:174.

48. Valecha N, Phyo AP, Mayxay M, Newton PN, Krudsood S, Keomany S, et al. An open-label, randomised study of dihydroartemisinin-piperaquine versus artesunate-mefloquine for falciparum malaria in Asia. PLoS One. 2010;5.

49. Borrmann S, Sasi P, Mwai L, Bashraheil M, Abdallah A, Muriithi S, et al. Declining responsiveness of plasmodium falciparum infections to Artemisinin-Based combination treatments on the Kenyan coast. PLoS One. 2011;6.

50. Sutanto I, Suprijanto S, Kosasih A, Dahlan MS, Syafruddin D, Kusriastuti R, et al. The effect of primaquine on gametocyte development and clearance in the treatment of uncomplicated falciparum malaria with dihydroartemisinin- piperaquine in South Sumatra, Western Indonesia: An open-label, randomized, controlled trial. Clin Infect Dis. 2013;56:685–93.

51. Ndiaye JLA, Faye B, Gueye A, Tine R, Ndiaye D, Tchania C, et al. Repeated treatment of recurrent uncomplicated Plasmodium falciparum malaria in Senegal with fixed-dose artesunate plus amodiaquine versus fixed-dose artemether plus lumefantrine: A randomized, open-label trial. Malar J. 2011;10:237.

52. Sirima SB, Ogutu B, Lusingu JPA, Mtoro A, Mrango Z, Ouedraogo A, et al. Comparison of artesunate-mefloquine and artemether-lumefantrine fixed-dose combinations for treatment of uncomplicated Plasmodium falciparum malaria in children younger than 5 years in sub-Saharan Africa: A randomised, multicentre, phase 4 trial. Lancet Infect Dis. 2016;16:1123–33.

53. Toure OA, Penali LK, Yapi JD, Ako BA, Toure W, Djerea K, et al. A comparative, randomized clinical trial of artemisinin/naphtoquine twice daily one day versus artemether/lumefantrine six doses regimen in children and adults with uncomplicated falciparum malaria in Cote d’Ivoire. Malar J. 2009;8:148.

54. Offianan AT, Assi SB, Coulibaly A, N’guessan LT, Ako AA, Kadjo FK, San MK PL. Assessment of the efficacy of first-line antimalarial drugs after 5 years of deployment by the National Malaria Control Programme in Côte d’Ivoire. Open Access J Clin Trials. 2011;3:Pages 67—76.

55. Yeka A, Lameyre V, Afizi K, Fredrick M, Lukwago R, Kamya MR, et al. Efficacy and safety of fixed-dose artesunate-amodiaquine vs. artemether-lumefantrine for repeated treatment of uncomplicated malaria in Ugandan children. PLoS One. Public Library of Science; 2014;9:e113311.

56. Carrara VI, Zwang J, Ashley EA, Price RN, Stepniewska K, Barends M, et al. Changes in the Treatment Responses to Artesunate-Mefloquine on the Northwestern Border of Thailand during 13 Years of Continuous Deployment. PLoS One. 2009;4:e4551.

57. Zongo I, Dorsey G, Rouamba N, Dokomajilar C, Sere Y, Rosenthal PJ, et al. Randomized Comparison of Amodiaquine plus Sulfadoxine-Pyrimethamine, Artemether-Lumefantrine, and Dihydroartemisinin-Piperaquine for the Treatment of Uncomplicated Plasmodium falciparum Malaria in Burkina Faso. Clin Infect Dis. 2007;45:1453–61.

58. Ursing J, Kofoed PE, Rodrigues A, Blessborn D, Thoft-Nielsen R, Björkman A, et al. Similar efficacy and tolerability of double-dose chloroquine and artemether-lumefantrine for treatment of Plasmodium falciparum infection in Guinea-Bissau: A randomized trial. J Infect Dis. 2011;203:109–16.

59. Martensson A, Ngasala B, Ursing J, Isabel Veiga M, Wiklund L, Membi C, et al. Influence of consecutive-day blood sampling on polymerase chain reaction-adjusted parasitological cure rates in an antimalarial-drug trial conducted in Tanzania. J Infect Dis. 2007;195:597–601.

60. Ashley EA, Lwin KM, McGready R, Simon WH, Phaiphun L, Proux S, et al. An open label randomized comparison of mefloquine-artesunate as separate tablets vs. a new co-formulated combination for the treatment of uncomplicated multidrug-resistant falciparum malaria in Thailand. Trop Med Int Heal. 2006;11:1653–60.

61. Kamya MR, Yeka A, Bukirwa H, Lugemwa M, Rwakimari JB, Staedke SG, et al. Artemether-lumefantrine versus dihydroartemisinin-piperaquine for treatment of malaria: A randomized trial. PLoS Clin Trials. 2007;2.

62. Ndiaye J, Randrianarivelojosia M, Sagara I, Brasseur P, Ndiaye I, Faye B, et al. Randomized, multicentre assessment of the efficacy and safety of ASAQ – a fixed-dose artesunate-amodiaquine combination therapy in the treatment of uncomplicated Plasmodium falciparum malaria. Malar J. 2009;8:125.

63. Karunajeewa HA, Mueller I, Senn M, Lin E, Law I, Gomorrai PS, et al. A Trial of Combination Antimalarial Therapies in Children from Papua New Guinea. N Engl J Med. 2008;359:2545–57.

64. Schramm B, Valeh P, Baudin E, Mazinda CS, Smith R, Pinoges L, et al. Tolerability and safety of artesunate-amodiaquine and artemether- lumefantrine fixed dose combinations for the treatment of uncomplicated Plasmodium falciparum malaria: Two open-label, randomized trials in Nimba County, Liberia. Malar J. 2013;12:250.

65. Bousema JT, Schneider P, Gouagna LC, Drakeley CJ, Tostmann A, Houben R, et al. Moderate effect of artemisinin-based combination therapy on transmission of Plasmodium falciparum. J Infect Dis. 2006;193:1151–9.

66. Yavo W, Faye B, Kuete T, Djohan V, Oga SA, Kassi RR, et al. Multicentric assessment of the efficacy and tolerability of dihydroartemisinin-piperaquine compared to artemether-lumefantrine in the treatment of uncomplicated Plasmodium falciparum malaria in sub-Saharan Africa. Malar J. BioMed Central Ltd; 2011;10:1–8.

67. Faye B, Ndiaye JL, Tine R, Sylla K, Gueye A, Lô AC, et al. A randomized trial of artesunate mefloquine versus artemether lumefantrine for the treatment of uncomplicated Plasmodium falciparum malaria in Senegalese children. Am J Trop Med Hyg. 2010;82:140–4.

68. Hwang J, Alemayehu BH, Hoos D, Melaku Z, Tekleyohannes SG, Teshi T, et al. In vivo efficacy of artemether-lumefantrine against uncomplicated Plasmodium falciparum malaria in Central Ethiopia. Malar J. 2011;10:209.

69. Ngasala BE, Malmberg M, Carlsson AM, Ferreira PE, Petzold MG, Blessborn D, et al. Efficacy and effectiveness of artemether-lumefantrine after initial and repeated treatment in children < 5 years of age with acute uncomplicated plasmodium falciparum malaria in rural Tanzania: A randomized trial. Clin Infect Dis. 2011;52:873–82.

70. Khadime Sylla, Annie Abiola, Roger Clément Kouly Tine, Babacar Faye, Doudou Sow, Jean Louis Ndiaye, Magatte Ndiaye, Aminata Colé LO, Kuaku Folly LAN and OG. Monitoring the efficacy and safety of three artemisinin combinations therapies (ACT) in Senegal: Results from two years surveillance. Am J Trop Med Hyg. 2013;89:251.

71. Bukirwa H, Yeka A, Kamya MR, Talisuna A, Banek K, Bakyaita N, et al. Artemisinin Combination Therapies for Treatment of Uncomplicated Malaria in Uganda. PLoS Clin Trials. 2006;1:e7.

72. Price RN, Uhlemann A-C, van Vugt M, Brockman A, Hutagalung R, Nair S, et al. Molecular and pharmacological determinants of the therapeutic response to artemether-lumefantrine in multidrug-resistant Plasmodium falciparum malaria. Clin Infect Dis. 2006;42:1570–7.

73. Faucher J-F, Aubouy A, Adeothy A, Cottrell G, Doritchamou J, Gourmel B, et al. Comparison of sulfadoxine-pyrimethamine, unsupervised artemether-lumefantrine, and unsupervised artesunate-amodiaquine fixed-dose formulation for uncomplicated plasmodium falciparum malaria in Benin: a randomized effectiveness noninferiority trial. J Infect Dis. 2009;200:57–65.

74. Mårtensson A, Strömberg J, Sisowath C, Msellem MI, Gil JP, Montgomery SM, et al. Efficacy of artesunate plus amodiaquine versus that of artemether-lumefantrine for the treatment of uncomplicated childhood Plasmodium falciparum malaria in Zanzibar, Tanzania. Clin Infect Dis. 2005;41:1079–86.

75. Collett D. Modelling Survival Data in Medical Research, Third Edition. Boca Raton: CRC Press Book; 2015.

76. Royston P, Parmar MKB. Flexible parametric proportional-hazards and proportional-odds models for censored survival data, with application to prognostic modelling and estimation of treatment effects. Stat Med. 2002;21:2175–97.
